# Supplementary material for: Cell-specific expression of key mitochondrial enzymes limits OXPHOS in astrocytes of the adult human neocortex and hippocampal formation
Source: Commun Biol. 2024 Aug 24;7:1045. doi: 10.1038/s42003-024-06751-z (PMC11344819; doi:10.1038/s42003-024-06751-z)
Supplement: Supplementary file 2 — Supplementary Information [file 42003_2024_6751_MOESM2_ESM.pdf]

Supplementary Table 1: Antibodies, vendors, RRIDs and their dilutions used in the present study.

**Protein against which antibody was raised**	**Vendor Cat no**	**Specific staining**	**RRID**	**Means of use (DAB only or double labelling with S100)**	**Dilution**
PDH E2	ab172617	Yes	AB_2827534	DAB only	1:200
PDH E1beta	ab155996	Yes	AB_2814826	Yes*	1:500
IDH2	CST 56439	Yes	AB_2799511	DAB only	1:100
IDH3A	MA5-25006	Yes	AB_2724500	fluorescent labelling	1:100
MDH2	CST 11908	Yes	AB_2797764	DAB only	1:100
SDHA	ab137040	No labelling	AB_2884996		
SDHB	ab175225	Yes	AB_2904585	DAB only	1:500
SDHC	ab155999	Yes	AB_2810989	DAB only	1:500
PDHX	ab155560	Yes	AB_2924648	DAB only	1:100
COX IV	ab14744	No labelling	AB_301443	-	
COX IV	CST 4850	Yes	AB_2085424	Yes*	1:500
Cyt b-c1 complex subunit 9	ab134909	No labelling	AB_2924649	-	
Cytochrome C	CST 11940	No labelling	AB_2637071	-	
Cytochrome c (6H2.B4)	CST 12963	Yes	AB_2637072	DAB only	1:100
Pyruvate carboxylase	16588-1-AP	Yes	AB_1851513	Yes*	1:500
MPC1	CST 14462	Yes	AB_2773729	Yes*	1:500
MPC2	CST 46141	Yes	AB_2799295	DAB only	1:100
alpha subunit ATPase	MS502	No labelling	AB_478268	-	
S100B	S2532	Yes	AB_477499		1:250
HuB+HuC+HuD	ab176106	Yes	AB_3099459	Yes**	1:200

* yes: double fluorescent labelling with S100B; **yes: double fluorescent labelling with COX IV

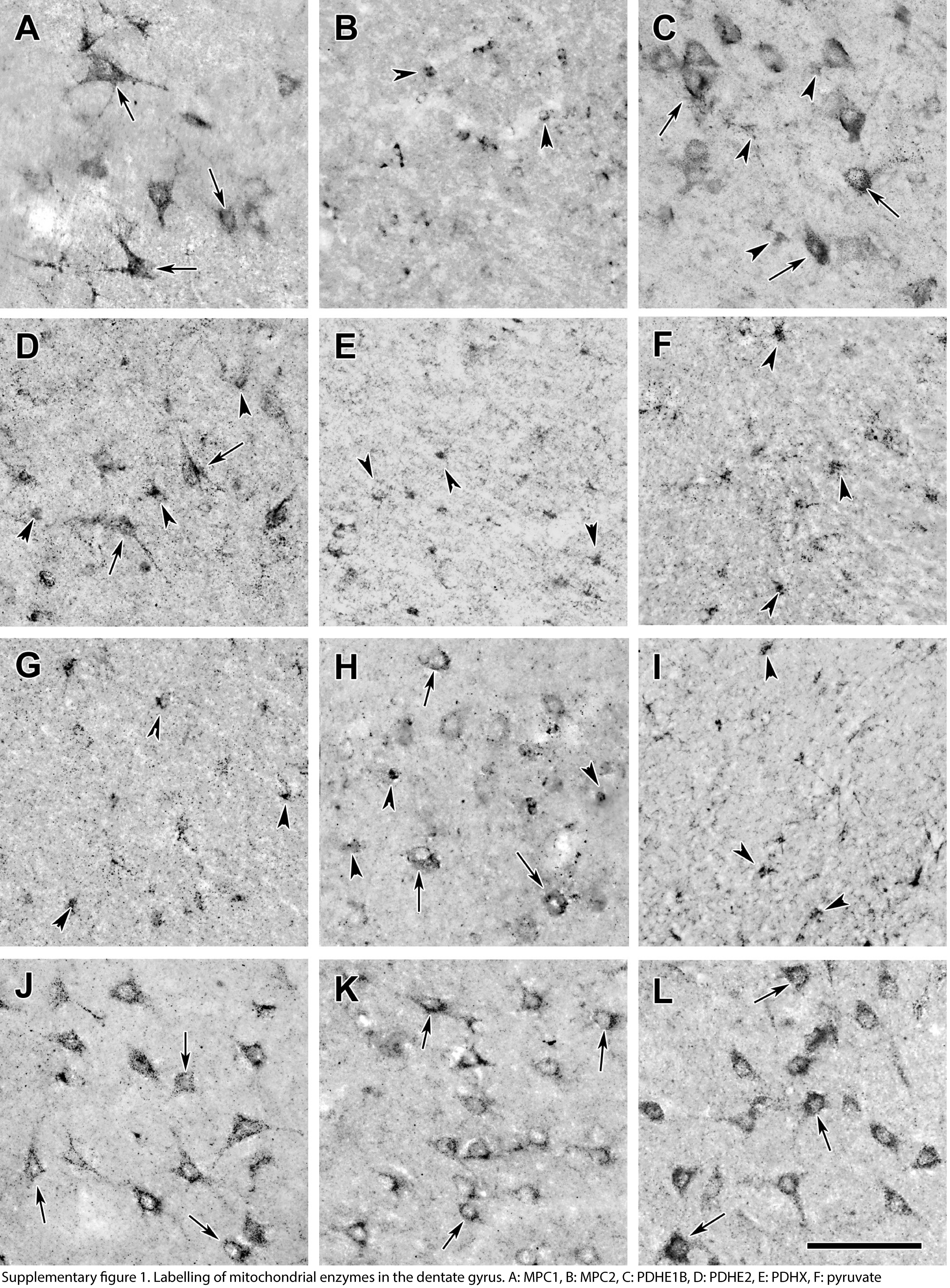

Supplementary figure 1. Labelling of mitochondrial enzymes in the dentate gyrus. A: MPC1, B: MPC2, C: PDHE1B, D: PDHE2, E: PDHX, F: pyruvate carboxylase, G: IDH2, H: SDH1B, I: SDH-C, J: MDH2, K: Cytochrome C, L: CoxIV. MPC1, MDH2, Cytochrome c and CoxIV, immunostaining labels exclusively neurons, while MPC2, PDHX, pyruvate carboxylase, IDH2 and SDH-C label only astrocytes. In turn, PDHE1B, PDHE2 and SDH1B immunoreactivity is present in both neuronal and glial cells. The magnification of all panels is equal. Scale bar = 100 μm.

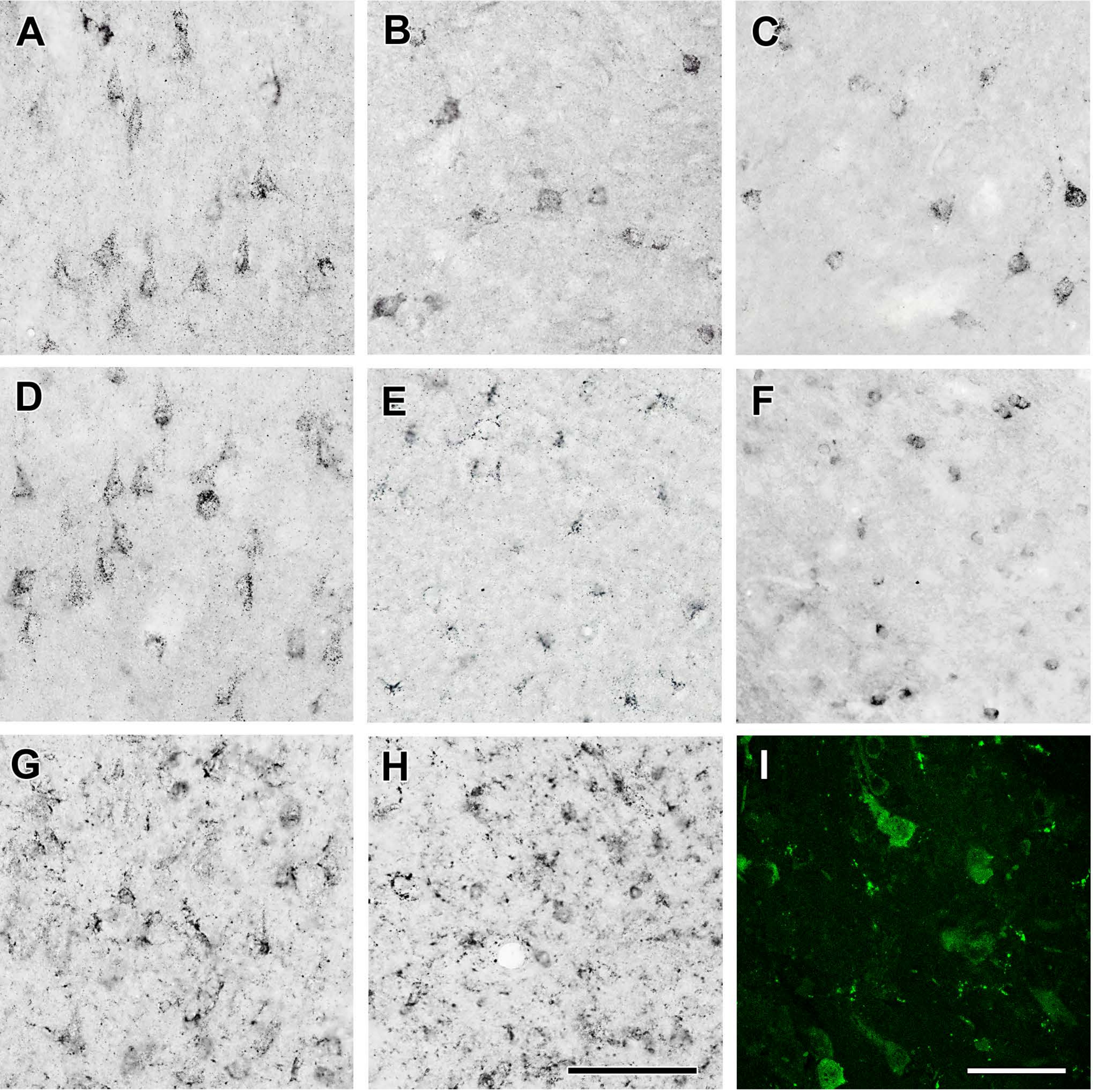

Supplementary figure 2. Labelling of selected mitochondrial enzymes in the parahippocampal gyrus. A: CoxIV, B: Cytochrome C, C: MPC1, D: MDH2, E: pyruvate carboxylase, F: MPC2, G: PDHE1B, H: SDH1B, I: IDH3A. CoxIV, Cytochrome C, MPC1, MDH2, and IDH3A immunostaining labels predominantly neurons (A-D, I), while pyruvate carboxylase and MPC2 (E, F) labels only astrocytes. In turn, PDHE1B and SDH1B immunoreactivity is present in both neuronal and glial cells (G, H). Scale bar = 100 μm for panels A-H, and 70 μm for panel I.

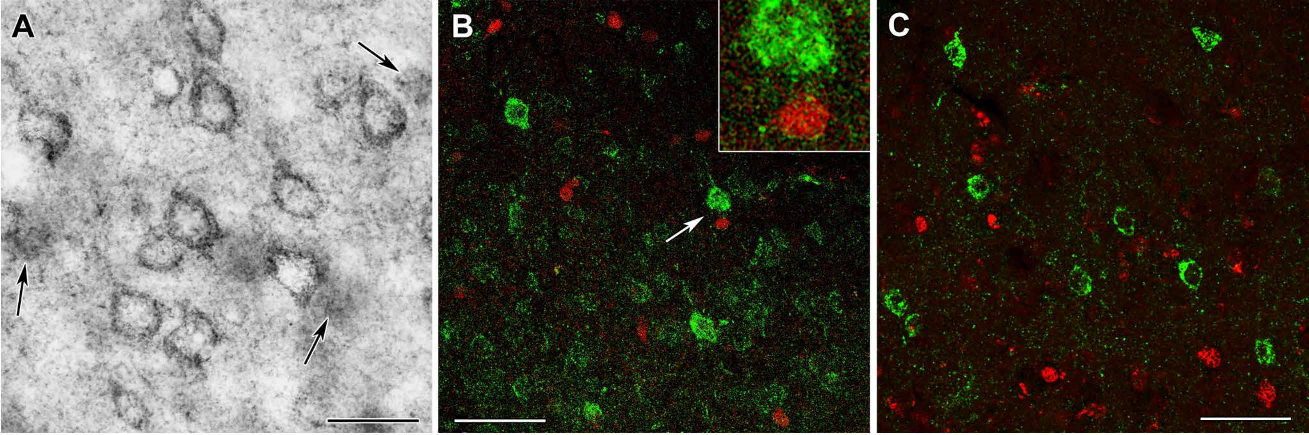

Supplementary figure 3. Neuronal and glial expression of CoxIV in the rat vs human cerebral cortex. A: DAB immunolabelled image shows several labelled neurons in the rat cerebral cortex. In addition, potential astrocytes, pointed to by black arrows, are also labelled. B: Double labelling of CoxIV and the astrocyte marker S100B in the rat cerebral cortex shows that in addition to intensely labelled neurons (green), S100B labelled astrocytes (red) also contain a moderate amount of CoxIV. The area indicated by the white arrow is enlarged in the inlet in the top right corner. C: Double labelling of CoxIV and the astrocyte marker S100B in the anterior cingulate cortex shows more selective neuronal expression of CoxIV in human. Scale bars = 30 μm for A, B and 60 μm for C.

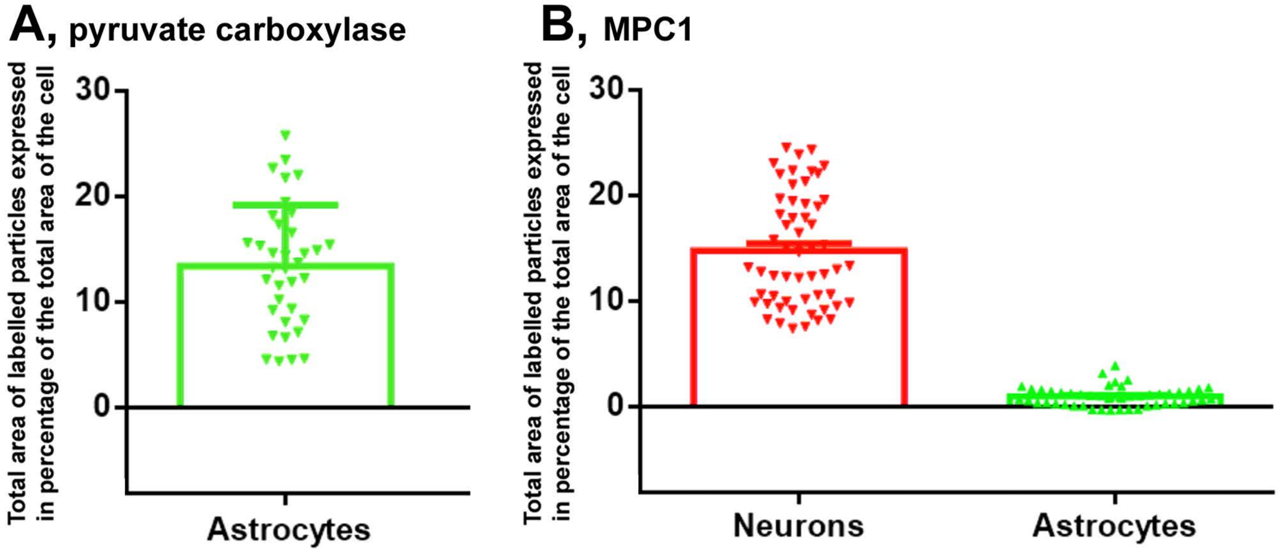

Supplementary figure 4. Quantification of the immunoreactivity of pyruvate carboxylase and MPC1 in neurons and astrocytes in the subiculum. A: The percentage of cell bodies covered by pyruvate carboxylase-immunoreactivity was high for astrocytes while neurons were not immunolabelled. B: In contrast, the percentage of cell bodies covered by MPC1 immunoreactivity was high for neurons while barely detectable in astrocytes.

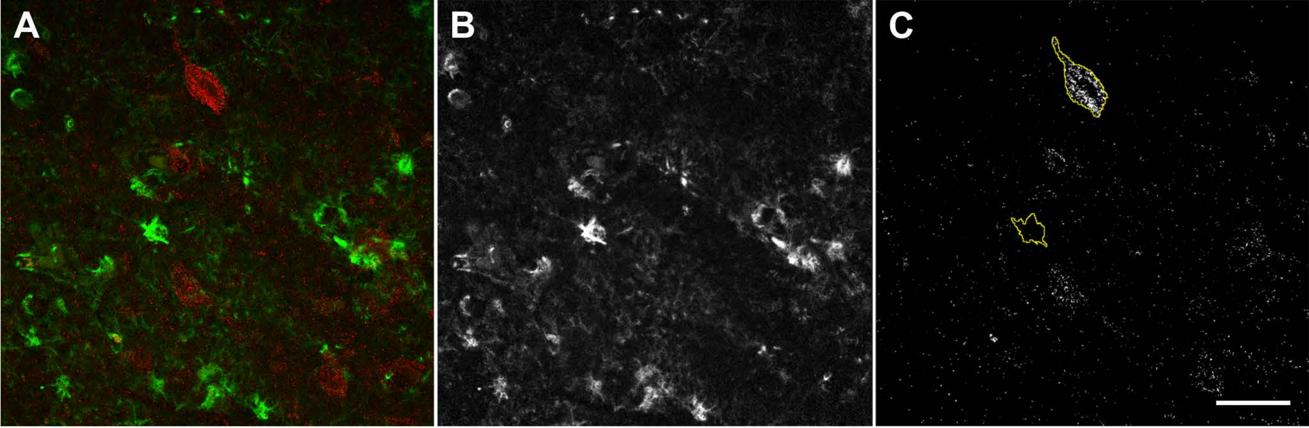

Supplementary figure 5. The method of quantification immunolabelling of neurons and astrocytes. A: An image of a section double labelled with CoxIV (red) and S100B (green). B: The green channel showing S100B, based on which the border of a selected cell was delineated. C: The mitochondrial marker is immunolabelled, based on which the borders of the cell body of a selected neuron is shown. The borders of the selected astrocyte is also shown with yellow line. The number of labelled pixels was counted within the delineated neurons and the delineated astrocytes.

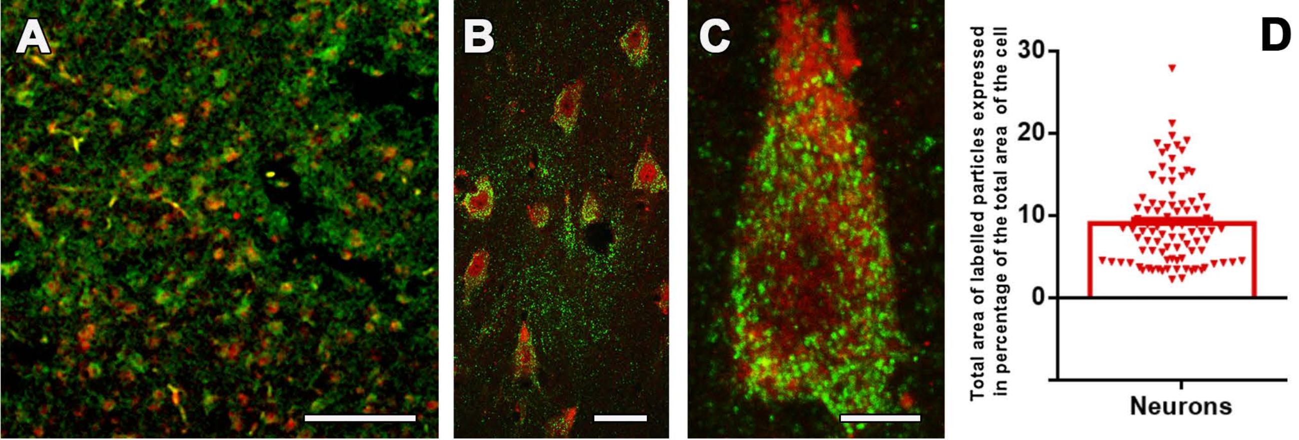

Supplementary figure 6. Neuronal expression of CoxIV in the dorsomedial prefrontal cortex determined in neurons identified by the selective neuronal marker HuC/D protein. CoxIV immunoreactivity is green while HuC/D immunoreactivity is red. A: A small magnification image shows the similar distribution of HuC/D and CoxIV-labelled neurons in all layers of the dorsomedial prefrontal cortex. B: A larger magnification image shows double labelling of all neuronal cell bodies. CoxIV-positive puncta are also located outside of the cell bodies in presumed presynaptic terminals. C: A large magnification photomicrograph indicating that HuC/D exhibits different subcellular location than CoxIV, the latter being present in mitochondria.
D: Quantification of CoxIV content of HuC/D -containing neurons (n=92). The total area of labelled mitochondria expressed in percentage of the total area of the cell was 9.06±0.54, a ratio almost identical to the one determined in neurons delineated by the mitochondrial marker (see main text).
Scale bars = 100 µm for A, 50 µm for B, and 10 µm for C.

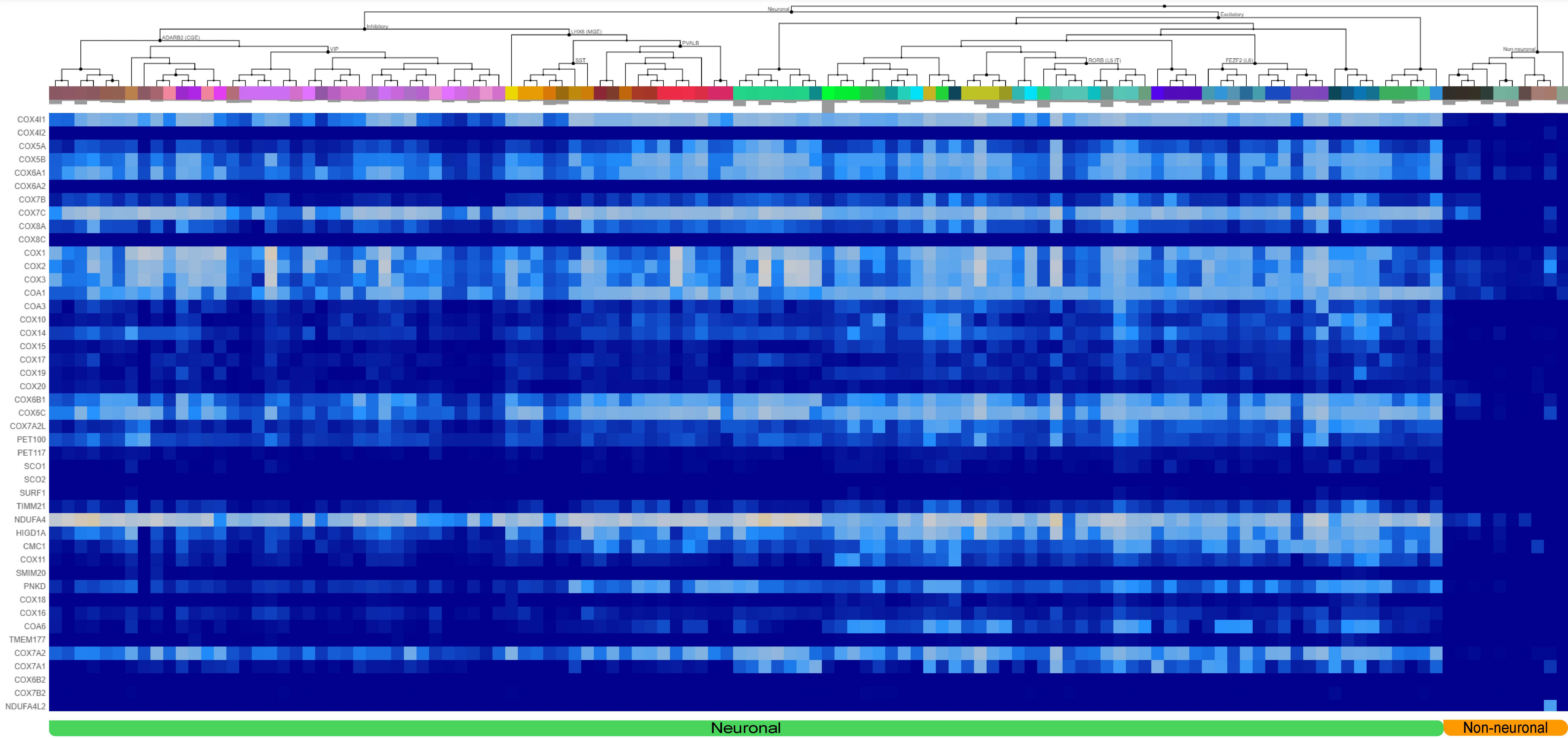

Supplementary figure 7: Heatmap of mRNA expression levels in neuronal and non-neuronal cells based on single cell sequencing by the Allen Institute for all subunits participating in Complex IV in human brain (homo) indicated on the y-axis. Queries were made by inputting gene symbols enlisted in MitoCarta 2.0 and curating it to include new data reported in MitoCarta 3.0. Heatmap range is identical to that shown in the last panel of Dataset homo depicting heatmap of homo Pi carriers and dicarboxylate carriers. On the top part of the x-axis, individual NeuN-positive nuclei (originating from neurons) vs NeuN-negative (originating from non-neuronal cells including astrocytes), segregated according to the clustering algorithm detailed in https://portal.brain-map.org/atlases-and-data/rnaseq/protocols-human-cortex are depicted. Cell clustering is outlined by the lines branching on the top of the x-axis. Abbreviations are given in https://portal.brain-map.org/atlases-and-data/rnaseq. Non-neuronal elements are shown in the far right of the heatmap, indicated as 'Non-neuronal' at the bottom (orange). Neuronal elements are indicated at the bottom of the heatmap in green.

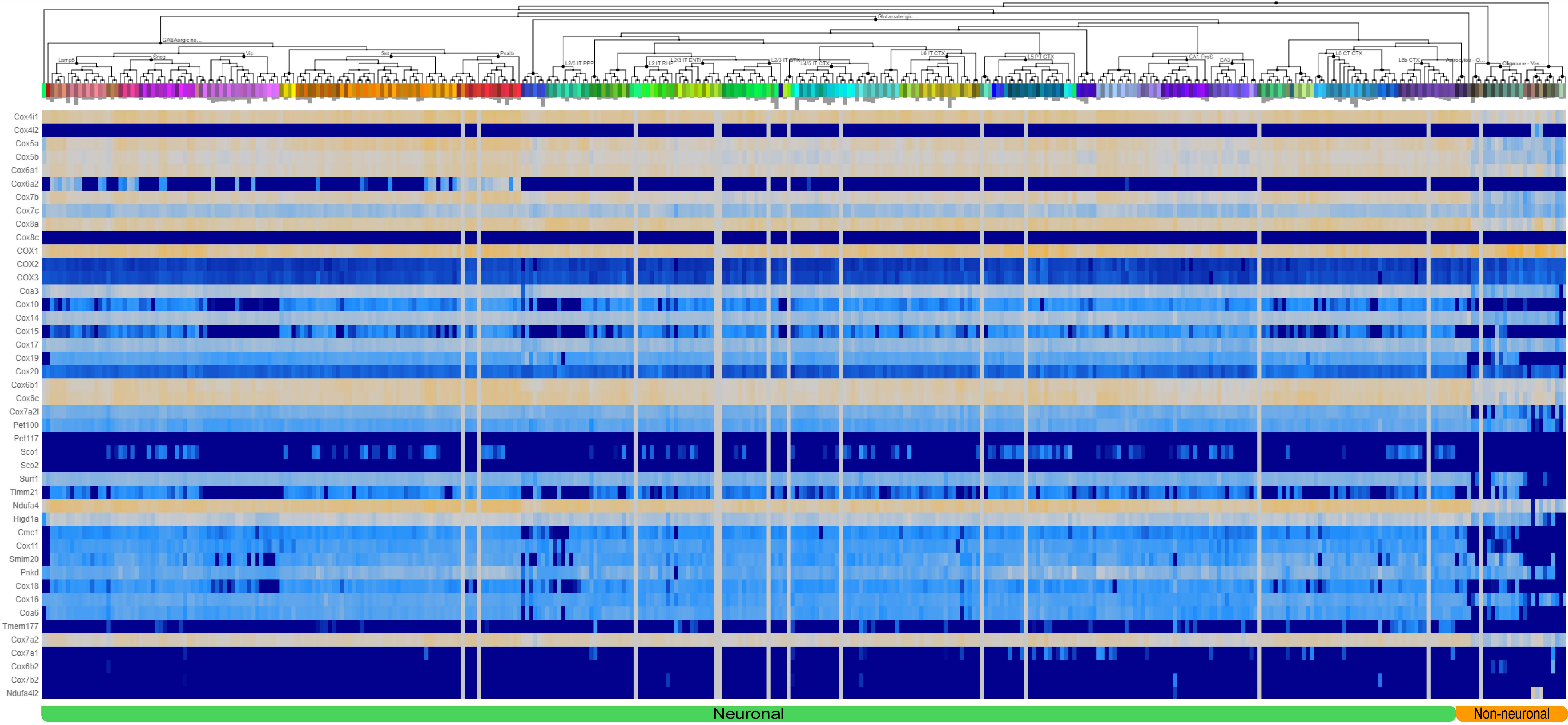

Supplementary figure 8: Heatmap of mRNA expression levels in neuronal and non-neuronal cells based on single cell sequencing by the Allen Institute for all subunits participating in Complex IV in mouse brain (mus) indicated on the y-axis. Queries were made by inputting gene symbols enlisted in MitoCarta 2.0 and curating it to include new data reported in MitoCarta 3.0. Heatmap range is identical to that shown in the last panel of Dataset mus depicting heatmap of mus Pi carriers and dicarboxylate carriers. On the top part of the x-axis, individual NeuN-positive nuclei (originating from neurons) vs NeuN-negative (originating from non-neuronal cells including astrocytes), segregated according to the clustering algorithm detailed in https://portal.brain-map.org/atlases-and-data/rnaseq/protocols-human-cortex are depicted. Cell clustering is outlined by the lines branching on the top of the x-axis. Abbreviations are given in https://portal.brain-map.org/atlases-and-data/rnaseq. Non-neuronal elements are shown in the far right of the heatmap, indicated as 'Non-neuronal' at the bottom (orange). Neuronal elements are indicated at the bottom of the heatmap in green.
