# Supplementary material for: Cell-specific expression of key mitochondrial enzymes limits OXPHOS in astrocytes of the adult human neocortex and hippocampal formation
Source: Commun Biol. 2024 Aug 24;7:1045. doi: 10.1038/s42003-024-06751-z (PMC11344819; doi:10.1038/s42003-024-06751-z)
Supplement: Supplementary file 3 — Description of Additional Supplementary Files [file 42003_2024_6751_MOESM3_ESM.pdf]

Description of Additional Supplementary Files

File name: Supplementary Data 1

Description: Source data for figure panels 1F3, 2F, 3E, 4D, 1A1, 1A2, 1A3, 6E, suppl 4A, suppl 4B, and suppl 6D.

File name: Supplementary Data 2

Description: Dataset homo and Dataset mus
