# Supplementary material for: Cell-specific expression of key mitochondrial enzymes limits OXPHOS in astrocytes of the adult human neocortex and hippocampal formation
Source: Commun Biol. 2024 Aug 24;7:1045. doi: 10.1038/s42003-024-06751-z (PMC11344819; doi:10.1038/s42003-024-06751-z)
Supplement: Supplementary file 4 — Supplementary Data 1 [file 42003_2024_6751_MOESM4_ESM.pdf]

		Neurons			
**Figure 1F3-Subiculum**		**Count**	**Total area (pixel^2)**	**Average size (pixel^2)**	**%Size**
Cox4S100-24x-4_z1	neuron1	181	2565	14.171	18.048
	neuron2	106	942	8.887	5.588
	neuron3	88	611	6.943	3.615
	neuron4	80	575	7.188	3.759
Cox4S100-24x-4_z2	neuron1	109	2266	20.789	16.699
	neuron2	184	1460	7.935	9.715
	neuron3	104	534	5.135	6.278
	neuron4	128	882	6.891	6.262
	neuron5	114	609	5.342	5.833
	neuron6	121	815	6.736	6.459
	neuron7	168	667	3.97	2.755
Cox4S100-24x-4_z3	neuron1	104	841	8.087	7.095
	neuron2	141	779	5.525	4.56
	neuron3	167	1225	7.335	7.07
	neuron4	74	533	7.203	5.297
	neuron5	194	1305	6.727	5.599
	neuron6	142	789	5.556	4.23
Cox4S100-80x-4_z1	neuron1	262	3446	13.153	7.34
	neuron2	236	3026	12.822	4.609
	neuron3	264	2701	10.231	4.01
	neuron4	474	5037	10.627	6.004
Cox4S100-80x-4_z2	neuron1	788	8794	11.16	19.217
	neuron2	1525	6992	4.585	10.787
	neuron3	474	5684	11.992	9.834
	neuron4	704	5145	7.308	6.315
	neuron5	539	11143	20.673	16.597
	neuron6	372	5773	15.519	12.638
	neuron7	840	7355	8.756	10.996
					**mean**
					**SEM**
**Figure 2F-DMPFC**					
	neuron1	320	1502	4.694	19.748
	neuron2	458	916	2	8.058
	neuron3	243	473	1.947	7.982
	neuron4	280	401	1.432	4.605
frontal-CoxIVS100-24x_z1	neuron1	646	2756	4.266	16.649
	neuron2	446	1725	3.868	17.281
	neuron3	273	510	1.868	7.979

	neuron4	374	831	2.222	9.783
frontal-CoxIVS100-24x_z2	neuron1	645	1817	2.817	12.476
	neuron2	347	870	2.507	8.869
	neuron3	294	437	1.486	6.243
	neuron4	339	465	1.372	4.178
	neuron5	231	327	1.416	4.616
frontal-CoxIVS100-24x_z6	neuron1	93	270	2.903	8.48
	neuron2	122	365	2.992	7.353
	neuron3	164	234	1.427	3.663
frontal-CoxIVS100-24x-2_z0	neuron1	347	682	1.965	5.269
	neuron2	139	263	1.892	4.023
frontal-CoxIVS100-24x-2_z2	neuron1	682	1237	1.814	7.715
	neuron2	454	660	1.454	5.699
frontal-CoxIVS100-24x-2_z3	neuron1	245	407	1.661	5.878
	neuron2	206	326	1.583	5.238
	neuron3	337	557	1.653	7.493
	neuron4	285	534	1.874	9.113
	neuron5	373	599	1.606	6.983
	neuron6	133	208	1.564	7.963
frontal-CoxIVS100-28x_z0	neuron1	423	1736	4.104	17.732
	neuron2	115	490	4.261	5.453
frontal-CoxIVS100-28x_z1	neuron1	182	1425	7.83	16.632
	neuron2	98	691	7.051	15.626
	neuron3	119	281	2.361	6.637
frontal-CoxIVS100-28x_z2	neuron1	430	1289	2.998	12.193
frontal-CoxIVS100-40x_z0	neuron1	483	2275	4.71	19.69
	neuron2	382	827	2.165	9.741
	neuron3	473	690	1.459	5.082
	neuron4	455	641	1.409	4.399
frontal-CoxIVS100-40x_z1	neuron1	478	3445	7.207	23.818

	neuron2	468	1315	2.81	10.866
	neuron3	576	1386	2.406	9.859
	neuron4	541	1453	2.686	11.861
frontal-CoxIVS100-40x_z2	neuron1	834	2372	2.844	13.133
	neuron2	577	880	1.525	6.027
	neuron3	655	2312	3.53	20.115
	neuron4	807	2017	2.499	11.858
frontal-CoxIVS100-40x_z3	neuron1	574	1741	3.033	15.32
	neuron2	662	1949	2.944	15.246
	neuron3	609	1020	1.675	6.424
frontal-CoxIVS100-40x_z4	neuron1	579	3844	6.639	24.475
	neuron2	474	1789	3.774	20.72
frontal-CoxIVS100-40x_z5	neuron1	672	2550	3.795	18.742
	neuron2	662	3880	5.861	22.561
	neuron3	492	1884	3.829	14.903
					**mean**
					**SEM**
**Figure suppl.4A-Subiculum**					
PyrcarbS100-24x-1_z2					
PyrcarbS100-24x-1_z3					
PyrcarbS100-24x-1_z4					

PyrcarbS100-24x-1_z5

Figure suppl. 4B-Subiculum

MPC1Iba1-24x-1_z0	neuron1	119	2225	18.697	15.259
	neuron2	63	3267	51.857	25.069
	neuron3	263	3069	11.669	17.517
	neuron4	94	1663	17.691	12.523
	neuron5	167	1547	9.263	10.238
	neuron6	160	1418	8.863	11.227
	neuron7	82	1704	20.78	21.663
MPC1Iba1-24x-1_z1	neuron1	347	3185	9.179	10.528
	neuron2	285	2199	7.716	10.215
	neuron3	266	2687	10.102	10.271
	neuron4	68	1636	24.059	19.716
	neuron5	97	3106	32.021	22.778
	neuron6	217	2245	10.346	11.325
	neuron7	128	1900	14.844	17.136
MPC1Iba1-24x-1_z2	neuron1	407	3611	8.872	11.193
	neuron2	260	2265	8.712	13.729
	neuron3	216	1223	5.662	8.353
	neuron4	251	2103	8.378	12.679
MPC1Iba1-24x-1_z3	neuron1	338	2081	6.157	7.853
	neuron2	236	1714	7.263	10.14
MPC1Iba1-24x-1_z4	neuron1	52	1305	25.096	17.97
MPC1Iba1-80x-1_z0	neuron1	501	8217	16.401	13.186
MPC1Iba1-80x-1_z1	neuron1	2117	24829	11.728	14.269
	neuron2	1236	23849	19.295	23.097

MPC1Iba1-80x-1_z2	neuron1	3613	36435	10.084	12.082
	neuron2	1969	18036	9.16	13.793
MPC1Iba1-80x-1_z3	neuron1	2421	19461	8.038	11.516
	neuron2	2864	27435	9.579	10.523
	neuron3	1516	11054	7.292	16.279
MPC1Iba1-80x-1_z4	neuron1	2638	24101	9.136	9.128
	neuron2	3649	32132	8.806	16.529
	neuron3	739	11178	15.126	20.009
MPC1Iba1-80x-1_z5	neuron1	1014	16277	16.052	18.466
MPC1S100-24x-1_z0	neuron1	122	1541	12.631	23.774
	neuron2	129	1307	10.132	20.839
MPC1S100-24x-1_z1	neuron1	122	1307	10.713	23.652
	neuron2	124	1371	11.056	20.487
	neuron3	143	1245	8.706	19.066
MPC1S100-24x-3_z1	neuron1	115	2789	24.252	18.641
	neuron2	198	2633	13.298	24.457
MPC1S100-24x-5legjobb_z1	neuron1	186	3849	20.694	22.987
	neuron2	313	3417	10.917	15.686
MPC1S100-24x-5legjobb_z4	neuron1	167	812	4.862	8.404
	neuron2	210	1400	6.667	12.449

MPC1S100-24xreal-1_z0	neuron1	181	2248	12.42	13.5
	neuron2	308	3653	11.86	21.375
	neuron3	282	2286	8.106	10.486
	neuron4	169	2691	15.923	19.43
MPC1S100-80x-5legjobb_z0	neuron1	1432	19365	13.523	12.945
	neuron2	976	11021	11.292	24.647
	neuron3	877	22526	25.685	23.021
MPC1S100-80x-5legjobb_z4	neuron1	1794	15936	8.883	9.851
	neuron2	1378	9320	6.763	8.813
	neuron3	2323	14023	6.037	8.3
MPC1S100-120x-5legjobb_z0	neuron1	2070	13876	6.703	8.747
MPC1S100-120x-5legjobb_z1	neuron1	7804	54036	6.924	15.408
					**mean**
					**SEM**

	Neurons				
**Figure 3E-Substantia nigra**		**Count**	**Total area (pixel^2)**	**Average size (pixel^2)**	**%Size**
SN-Cox4S100-24x-1_z1m	neuron1	223	3050	13.677	35.832
	neuron2	196	2621	13.372	36.042
	neuron3	211	2715	12.867	36.869
	neuron4	112	2550	22.768	48.794
SN-Cox4S100-24x-1_z2m	neuron1	304	17060	56.118	61.473
	neuron2	160	6382	39.888	56.478
	neuron3	248	4175	16.835	38.816
	neuron4	181	3831	21.166	47.864
	neuron5	327	7088	21.676	45.395
SN-Cox4S100-24x-1_z3m	neuron1	135	6159	45.622	53.584
	neuron2	243	8421	34.654	52.823
	neuron3	226	6773	29.969	52.602
	neuron4	180	4166	23.144	48.307

	neuron5	361	15476	42.87	58.032
	neuron6	395	4665	11.81	35.096
SN-Cox4S100-24x-1_z4m	neuron1	146	5684	38.932	54.34
	neuron2	537	4961	9.238	29.771
	neuron3	187	11543	61.727	62.932
SN-Cox4S100-24x-1_z5m	neuron1	181	5282	29.182	46.918
	neuron2	334	7744	23.186	49.788
	neuron3	484	3724	7.694	26.097
SN-Cox4S100-24x-2_z0m	neuron1	369	6593	17.867	37.614
	neuron2	227	4954	21.824	37.564
	neuron3	244	8059	33.029	49.284
	neuron4	280	4748	16.957	35.528
	neuron5	353	8428	23.875	37.652
SN-Cox4S100-24x-2_z1m	neuron1	266	7425	27.914	46.112
	neuron2	104	2773	26.663	48.462
	neuron3	268	5314	19.828	43.07
	neuron4	69	4507	65.319	55.341
	neuron5	488	7988	16.369	38.118
SN-Cox4S100-24x-2_z2m	neuron1	344	6591	19.16	35.762
	neuron2	288	4465	15.503	35.589
	neuron3	221	3381	15.299	34.269
	neuron4	919	10696	11.639	30.511
SN-Cox4S100-24x-2_z3m	neuron1	277	5821	21.014	40.228
	neuron2	363	3948	10.876	27.345
	neuron3	665	6963	10.471	28.652
	neuron4	586	5520	9.42	29.547
	neuron5	1181	12559	10.634	27.436
SN-Cox4S100-24x-2_z4m	neuron1	417	4700	11.271	30.909
	neuron2	377	4543	12.05	31.707
	neuron3	453	3579	7.901	24.71
SN-Cox4S100-24x-2_z5m	neuron1	418	3068	7.34	24.272
	neuron2	579	4003	6.914	23.467
SN-Cox4S100-24x-3_z0m	neuron1	222	9446	42.55	55.066
SN-Cox4S100-24x-3_z1m	neuron1	364	11601	31.871	47.409
SN-Cox4S100-24x-3_z2m	neuron1	588	10172	17.299	35.351
	neuron2	458	3877	8.465	26.954

	neuron3	546	4843	8.87	26.476
SN-Cox4S100-24x-3_z3m	neuron1	601	7326	12.19	33.569
SN-Cox4S100-24x-3_z4m	neuron1	486	6238	12.835	32.258
SN-Cox4S100-24x-4_z0m	neuron1	684	11580	16.93	39.348
	neuron2	349	4593	13.16	28.233
	neuron3	318	5076	15.962	32.643
SN-Cox4S100-24x-4_z1m	neuron1	344	7484	21.756	42.947
	neuron2	456	5788	12.693	24.596
	neuron3	315	3443	10.93	30.345
	neuron4	395	4940	12.506	33.311
SN-Cox4S100-24x-4_z2m	neuron1	350	7778	22.223	45.432
	neuron2	379	4735	12.493	34.542
SN-Cox4S100-24x-4_z3m	neuron1	464	4304	9.276	26.467
	neuron2	298	4341	14.567	39.507
SN-Cox4S100-24x-4_z4m	neuron1	282	4369	15.493	39.747
					**mean**
					**SEM**

**Figure suppl. 6D**	**Neurons**				
		**Count**	**Total area (pixel^2)**	**Average size (pixel^2)**	**%Size**
CoxIV+Hubcd-nagy szelet-24x-1_z0	neuron1	283	2281	8.06	12.561
	neuron2	121	3601	29.76	29.064
	neuron3	139	1937	13.935	19.432
	neuron4	98	1623	16.561	16.568
	neuron5	166	1374	8.277	12.507
	neuron6	145	956	6.593	9.309
CoxIV+Hubcd-nagy szelet-24x-1_z1	neuron1	165	2310	14	19.586
	neuron2	123	538	4.374	3.722
	neuron3	339	3545	10.457	21.642
	neuron4	323	1610	4.985	9.082
	neuron5	117	406	3.47	3.974
	neuron6	216	1591	7.366	14.683
CoxIV+Hubcd-nagy szelet-24x-1_z2	neuron1	296	744	2.514	4.832
	neuron2	288	1454	5.049	8.286
	neuron3	289	701	2.426	4.549
	neuron4	137	549	4.007	3.528

	neuron5	460	3265	7.098	16.66
	neuron6	411	3058	7.44	19.969
	neuron7	245	538	2.196	3.414
	neuron8	322	1589	4.935	9.273
CoxIV+Hubcd-nagy szelet-24x-1_z3	neuron1	302	727	2.407	4.971
	neuron2	347	934	2.692	5.558
	neuron3	433	3294	7.607	20.954
	neuron4	465	3298	7.092	19.817
CoxIV+Hubcd-nagy szelet-24x-1_z4	neuron1	214	771	3.603	4.755
CoxIV+Hubcd-nagy szelet-24x-2_z0	neuron1	84	691	8.226	5.004
	neuron2	140	1371	9.793	7.577
	neuron3	80	1435	17.938	14.831
CoxIV+Hubcd-nagy szelet-24x-2_z1	neuron1	153	2573	16.817	15.545
	neuron2	80	1321	16.512	9.702
	neuron3	91	1041	11.44	8.873
	neuron4	118	1913	16.212	12.502
	neuron5	114	2064	18.105	11.509
	neuron6	184	1516	8.239	11.79
	neuron7	106	826	7.792	6.937
	neuron8	66	948	14.364	9.371
CoxIV+Hubcd-nagy szelet-24x-2_z2	neuron1	130	1348	10.369	9.799
	neuron2	164	2714	16.549	17.404
	neuron3	158	2266	14.342	12.974
	neuron4	167	1766	10.575	11.971
	neuron5	163	1976	12.123	9.816
	neuron6	162	1489	9.191	9.517
	neuron7	148	1044	7.054	10.302
	neuron8	226	1102	4.876	6.066
	neuron9	147	3182	21.646	19.129
CoxIV+Hubcd-nagy szelet-24x-2_z3	neuron1	147	1221	8.306	10.819
	neuron2	178	1806	10.146	11.824
	neuron3	190	1478	7.779	8.054
	neuron4	153	1062	6.941	6.989
	neuron5	184	2132	11.587	9.225
	neuron6	165	861	5.218	5.436
	neuron7	250	3728	14.912	16.136
CoxIV+Hubcd-nagy szelet-24x-2_z4	neuron1	127	661	5.205	4.172
	neuron2	225	1522	6.764	6.709
	neuron3	292	1932	6.616	11.835
	neuron4	75	520	6.933	4.111

CoxIV+Hubcd-nagy szelet-24x-2_z5	neuron1	210	1272	6.057	6.073
	neuron2	129	658	5.101	3.739
	neuron3	283	1914	6.763	12.562
CoxIVHubcd-nagy szelet-24x-3_z0	neuron1	165	2164	13.115	17.221
	neuron2	204	2001	9.809	8.74
	neuron3	137	916	6.686	10.825
	neuron4	98	735	7.5	7.644
	neuron5	137	918	6.701	10.11
	neuron6	110	851	7.736	6.004
	neuron7	104	1436	13.808	12.735
	neuron8	136	950	6.985	5.81
CoxIVHubcd-nagy szelet-24x-3_z1	neuron1	234	2036	8.701	10.252
	neuron2	293	2887	9.853	12.843
	neuron3	143	2906	20.322	18.536
	neuron4	141	1388	9.844	11.261
	neuron5	142	1071	7.542	7.473
	neuron6	135	719	5.326	4.084
CoxIVHubcd-nagy szelet-24x-3_z2	neuron1	224	1649	7.362	7.543
	neuron2	164	1126	6.866	6.778
	neuron3	183	870	4.754	5.811
	neuron4	202	2599	12.866	16.323
	neuron5	141	1177	8.348	9.932
	neuron6	269	1561	5.803	8.304
	neuron7	190	952	5.011	4.531
CoxIVHubcd-nagy szelet-24x-3_z3	neuron1	204	1002	4.912	5.529
	neuron2	205	2089	10.19	11.785
	neuron3	174	1005	5.776	5.342
	neuron4	154	692	4.494	4.27
	neuron5	192	1366	7.115	8.176
	neuron6	182	997	5.478	5.151
	neuron7	262	1119	4.271	4.952
CoxIVHubcd-nagy szelet-24x-3_z4	neuron1	170	674	3.965	3.573
	neuron2	181	1477	8.16	7.997
	neuron3	183	753	4.115	3.672
	neuron4	147	728	4.952	4.794
CoxIVHubcd-nagy szelet-24x-3_z5	neuron1	171	815	4.766	4.758
					**mean**
					**SEM**

Neurons

Figure 4D-CA1/CA3 region of the hippocampus		Count	Total area (pixel^2)	Average size (pixel^2)	%Size
20174963-CA1	neuron1	12	44	3.667	7.871
	neuron2	8	9	1.125	2.027
	neuron3	9	16	1.778	3.67
	neuron4	4	60	15	15.789
	neuron5	11	14	1.273	3.03
20174963-CA1	neuron1	28	59	2.107	7.143
	neuron2	4	9	2.25	1.528
	neuron3	22	62	2.818	8.267
	neuron4	9	24	2.667	3.871
	neuron5	18	36	2	4.694
20174963-CA3	neuron1	23	46	2	7.348
	neuron2	1	1	1	0.199
	neuron3	20	56	2.8	8.321
	neuron4	17	45	2.647	6.484
	neuron5	15	27	1.8	4.32
20174963-CA3	neuron1	9	10	1.111	1.193
	neuron2	19	28	1.474	4.076
	neuron3	22	106	4.818	7.91
	neuron4	19	26	1.368	2.577
	neuron5	10	17	1.7	2.163
20174964-CA3	neuron1	12	32	2.667	7.459
	neuron2	12	16	1.333	3.509
	neuron3	13	22	1.692	3.748
	neuron4	10	42	4.2	8.14
	neuron5	8	12	1.5	2.626
20174964-CA1	neuron1	11	26	2.364	5.231
	neuron2	4	14	3.5	2.201
	neuron3	15	95	6.333	10.759
	neuron4	17	52	3.059	7.602
	neuron5	14	47	3.357	6.096
20174964-CA1	neuron1	11	15	1.364	2.427
	neuron2	26	50	1.923	6.588
	neuron3	19	49	2.579	5.799
	neuron4	9	10	1.111	1.285
	neuron5	14	24	1.714	3.577
20174964-CA1	neuron1	17	35	2.059	4.795
	neuron2	14	48	3.429	7.251

	neuron3	13	20	1.538	2.326
	neuron4	12	19	1.583	2.746
	neuron5	18	34	1.889	4.282
20174964-CA3	neuron1	3	4	1.333	1.266
	neuron2	3	5	1.667	1.25
	neuron3	5	8	1.6	1.626
	neuron4	8	8	1	1.79
	neuron5	5	5	1	0.958
20185944-CA3	neuron1	10	19	1.9	2.497
	neuron2	11	51	4.636	8.333
	neuron3	8	8	1	1.351
	neuron4	8	21	2.625	3.125
	neuron5	10	17	1.7	2.475
20185944-CA3	neuron1	8	19	2.375	4.077
	neuron2	7	11	1.571	2.331
	neuron3	11	16	1.455	2.532
	neuron4	13	23	1.769	3.555
	neuron5	9	20	2.222	2.849
20185944-CA3	neuron1	11	64	5.818	10.561
	neuron2	15	61	4.067	9.091
	neuron3	21	39	1.857	6.094
	neuron4	15	26	1.733	4.924
	neuron5	5	38	7.6	6.597
20185944-CA1	neuron1	25	97	3.88	11.162
	neuron2	23	261	11.348	15.21
	neuron3	19	27	1.421	3.534
	neuron4	29	51	1.759	4.793
	neuron5	21	48	2.286	4.974
20185944-CA1	neuron1	9	15	1.667	2.366
	neuron2	10	14	1.4	1.774
	neuron3	9	12	1.333	1.521
	neuron4	17	19	1.118	2.328
	neuron5	13	20	1.538	2.484
20185947-CA3	neuron1	12	16	1.333	1.784
	neuron2	12	17	1.417	1.736
	neuron3	28	141	5.036	9.86
	neuron4	30	109	3.633	9.758
	neuron5	40	154	3.85	12.582
20185947-CA3	neuron1	12	38	3.167	3.201

	neuron2	21	34	1.619	3.172
	neuron3	13	20	1.538	2.075
	neuron4	35	67	1.914	7.001
	neuron5	18	32	1.778	2.038
20185947-CA1	neuron1	13	240	18.462	23.553
	neuron2	20	37	1.85	2.384
	neuron3	9	14	1.556	1.39
	neuron4	19	197	10.368	18.241
	neuron5	14	35	2.5	3.553
20185947-CA1	neuron1	7	13	1.857	1.015
	neuron2	4	5	1.25	0.446
	neuron3	9	140	15.556	21.472
	neuron4	6	10	1.667	1.49
	neuron5	20	139	6.95	13.101
20185951-CA3	neuron1	17	22	1.294	1.712
	neuron2	23	56	2.435	5.639
	neuron3	33	56	1.697	4.358
	neuron4	13	35	2.692	3.117
	neuron5	16	21	1.312	2.156
20185951-CA3	neuron1	35	221	6.314	17.909
	neuron2	15	73	4.867	7.249
	neuron3	26	115	4.423	11.523
	neuron4	14	89	6.357	9.319
	neuron5	28	141	5.036	13.252
20185951-CA1	neuron1	26	72	2.769	9.137
	neuron2	10	180	18	23.684
	neuron3	11	182	16.545	21.412
	neuron4	9	116	12.889	16.044
	neuron5	31	97	3.129	10.168
20185951-CA1	neuron1	15	153	10.2	17.688
	neuron2	18	85	4.722	6.625
	neuron3	10	23	2.3	2.561
	neuron4	5	12	2.4	1.412
	neuron5	11	31	2.818	3.605
20196904-CA3	neuron1	4	4	1	0.613
	neuron2	8	8	1	1.254
	neuron3	12	13	1.083	1.487
	neuron4	2	3	1.5	0.407
	neuron5	1	1	1	0.126

20196904-CA1	neuron1	4	5	1.25	1.397
	neuron2	4	6	1.5	1.471
	neuron3	4	7	1.75	1.42
	neuron4	4	7	1.75	1.728
	neuron5	4	5	1.25	0.996
20196904-CA1	neuron1	12	22	1.833	4.857
	neuron2	9	23	2.556	4.946
	neuron3	20	36	1.8	5.634
	neuron4	9	29	3.222	6.488
	neuron5	13	29	2.231	5.513
20196907-CA1	neuron1	17	24	1.412	2.771
	neuron2	3	3	1	0.413
	neuron3	14	17	1.214	1.965
	neuron4	15	25	1.667	2.994
	neuron5	9	10	1.111	1.105
20196907-CA1	neuron1	1	1	1	0.267
	neuron2	3	3	1	0.437
	neuron3	2	2	1	0.357
	neuron4	2	2	1	0.27
	neuron5	5	5	1	0.688
20196907-CA3	neuron1	12	18	1.5	2.49
	neuron2	12	17	1.417	2.482
	neuron3	15	29	1.933	4.108
	neuron4	11	17	1.545	2.24
	neuron5	11	11	1	1.366
20196907-CA3	neuron1	11	20	1.818	2.395
	neuron2	26	53	2.038	6.471
	neuron3	20	31	1.55	3.229
	neuron4	12	16	1.333	2.572
	neuron5	16	19	1.188	2.311
20196907-CA3	neuron1	21	25	1.19	2.165
	neuron2	17	36	2.118	4.952
	neuron3	6	11	1.833	1.449
	neuron4	10	14	1.4	1.617
	neuron5	17	25	1.471	2.907
20196924-CA3	neuron1	4	4	1	0.607
	neuron2	15	30	2	3.968
	neuron3	18	62	3.444	8.832
	neuron4	17	42	2.471	4.839
	neuron5	19	29	1.526	2.956

20196924-CA3	neuron1	16	40	2.5	4.479
	neuron2	15	120	8	13.029
	neuron3	11	36	3.273	4.768
	neuron4	13	29	2.231	2.816
	neuron5	6	10	1.667	1.757
20196924-CA1	neuron1	12	65	5.417	6.959
	neuron2	5	12	2.4	1.788
	neuron3	5	11	2.2	1.189
	neuron4	29	129	4.448	11.257
	neuron5	15	25	1.667	3.383
20196944-CA1	neuron1	19	39	2.053	4.994
	neuron2	13	16	1.231	1.556
	neuron3	37	84	2.27	6.965
	neuron4	31	57	1.839	5.858
	neuron5	18	62	3.444	7.135
20196944-CA1	neuron1	31	49	1.581	5.765
	neuron2	58	156	2.69	10.236
	neuron3	31	88	2.839	10.745
	neuron4	25	97	3.88	15.156
	neuron5	39	99	2.538	9.244
20196944-CA3	neuron1	25	31	1.24	2.989
	neuron2	50	77	1.54	5.453
	neuron3	30	39	1.3	4.402
	neuron4	20	54	2.7	6.421
	neuron5	10	18	1.8	2.4
20196944-CA3	neuron1	14	26	1.857	2.832
	neuron2	10	10	1	1.292
	neuron3	19	27	1.421	3.936
	neuron4	17	24	1.412	3.077
	neuron5	8	9	1.125	0.975
20207903-CA3	neuron1	31	99	3.194	9.792
	neuron2	32	83	2.594	8.25
	neuron3	35	87	2.486	9.295
	neuron4	22	76	3.455	8.736
	neuron5	33	99	3	8.616
20207903-CA3	neuron1	65	142	2.185	10.581
	neuron2	43	76	1.767	7.245
	neuron3	46	120	2.609	10.563
	neuron4	37	76	2.054	6.947

	neuron5	55	151	2.745	13.47
20207903-CA1	neuron1	21	68	3.238	9.855
	neuron2	24	93	3.875	8.151
	neuron3	25	54	2.16	6.193
	neuron4	29	56	1.931	7.134
	neuron5	18	30	1.667	4.196
20207903-CA1	neuron1	24	30	1.25	4.027
	neuron2	15	36	2.4	6.294
	neuron3	16	25	1.562	3.289
	neuron4	27	40	1.481	4.43
	neuron5	18	54	3	5.666

		Astrocytes						
		**Count**	**Total area (pixel^2)**	**Average size (pixel^2)**	**%Size**			**Count**
18.022	astrocyte1	5	7	1.4	0.096	0.07	control1	3
5.546	astrocyte2	7	28	4	0.469	0.427	control2	3
3.576	astrocyte3	9	14	1.556	0.166	0.127	control3	2
3.708	astrocyte4	4	5	1.25	0.106	0.055	control4	4
16.58	astrocyte1	25	47	1.88	0.874	0.755	control1	4
9.374	astrocyte2	19	26	1.368	0.424	0.083	control2	11
6.218	astrocyte3	15	25	1.667	0.341	0.281	control3	2
5.609	astrocyte4	10	19	1.9	0.419	-0.234	control4	12
5.572	astrocyte5	5	5	1	0.113	-0.148	control5	9
5.889	astrocyte6	11	29	2.636	0.542	-0.028	control6	15
2.103	astrocyte7	9	150	16.667	3.952	3.3	control7	15
	astrocyte8	2	3	1.5	0.089	0.089		
7.049							control1	2
4.18							control2	9
6.97							control3	3
4.793							control4	9
4.871							control5	26
3.879							control6	5
6.86	astrocyte1	70	232	3.314	0.469	-0.011	control1	36
4.292	astrocyte2	77	154	2	0.443	0.126	control2	26
4.01								
6.004								
18.714	astrocyte1	326	1167	3.58	1.106	0.603	control1	146
9.984	astrocyte2	158	579	3.665	1.102	0.299	control2	74
9.122	astrocyte3	86	390	4.535	0.622	-0.09	control3	79
5.899	astrocyte4	37	167	4.514	0.51	0.094	control4	38
16.597								
12.638								
10.996								
**7.823393**						**0.322111**		
**0.883766**						**0.184851**		
17.56	astrocyte1	77	107	1.39	4.477	2.289	control1	60
4.411	astrocyte2	66	78	1.182	2.718	-0.929	control2	69
6.259	astrocyte3	63	88	1.397	4.867	3.144	control3	46
3.546		137	211	1.54	4.378	3.319	control4	32
15.004	astrocyte1	70	79	1.129	3.382	1.737	control1	50
15.956	astrocyte2	91	108	1.187	3.843	2.518	control2	72
7.031	astrocyte3	92	117	1.272	4.483	3.535	control3	44

8.204	astrocyte4	19	26	1.368	1.318	-0.261	control4	66
	astrocyte5	44	45	1.023	1.601	0.039	control5	76
10.737	astrocyte1	70	79	1.129	2.537	0.798	control1	54
8.101	astrocyte2	31	35	1.129	1.222	0.454	control2	14
5.371	astrocyte3	45	50	1.111	2.281	1.409	control3	24
3.485	astrocyte4	27	27	1	1.458	0.765	control4	36
4.363	astrocyte5	38	47	1.237	2.031	1.778	control5	11
7.885	astrocyte1	39	41	1.051	1.975	1.38	control1	19
7.063	astrocyte2	35	37	1.057	1.36	1.07	control2	7
2.769	astrocyte3	21	22	1.048	0.84	-0.054	control3	27
4.753	astrocyte1	50	54	1.08	0.708	0.192	control1	42
3.067	astrocyte2	65	66	1.015	1.058	0.102	control2	65
6.789	astrocyte1	124	150	1.21	2.824	1.898	control1	72
3.916	astrocyte2	73	117	1.603	2.796	1.013	control2	172
3.132	astrocyte1	97	121	1.247	4.028	1.282	control1	135
2.731	astrocyte2	76	87	1.145	3.114	0.607	control2	123
5.793	astrocyte3	84	110	1.31	4.307	2.607	control3	107
6.367								
4.476								
6.263								
17.096	astrocyte1	34	47	1.382	1.784	1.148	control1	13
5.227	astrocyte2	18	20	1.111	1.058	0.832	control2	11
	astrocyte3	34	81	2.382	3.347	3.081	control3	13
	astrocyte4	2	2	1	0.145	-0.547	control4	36
16.315	astrocyte1	21	28	1.333	0.607	0.29	control1	13
15.567	astrocyte2	17	19	1.118	0.958	0.899	control2	3
6.471	astrocyte3	35	51	1.457	1.865	1.699	control3	10
	astrocyte4	26	35	1.346	1.113	0.872	control4	9
12.141	astrocyte1	14	14	1	0.366	0.314	control1	3
	astrocyte2	10	10	1	0.213	0.179	control2	2
	astrocyte3	44	62	1.409	1.572	1.449	control3	8
	astrocyte4	11	11	1	0.364	0.281	control4	5
19.054	astrocyte1	69	74	1.072	1.8	1.164	control1	18
8.439	astrocyte2	31	32	1.032	0.94	-0.362	control2	35
3.544	astrocyte3	64	77	1.203	2.318	0.78	control3	49
2.919	astrocyte4	38	42	1.105	2.819	1.339	control4	48
22.988	astrocyte1	113	131	1.159	2.924	2.094	control1	29

8.738	astrocyte2	116	154	1.328	4.413	2.285	control2	106
8.686	astrocyte3	51	65	1.275	2.041	0.868	control3	79
10.841	astrocyte4	99	109	1.101	2.514	1.494	control4	63
11.898	astrocyte1	76	85	1.118	2.459	1.224	control1	47
4.466	astrocyte2	93	102	1.097	2.71	1.149	control2	79
19.184	astrocyte3	72	77	1.069	2.089	1.158	control3	61
10.485	astrocyte4	53	63	1.189	2.925	1.552	control4	63
14.572	astrocyte1	75	81	1.08	1.611	0.863	control1	50
13.953	astrocyte2	36	37	1.028	0.978	-0.315	control2	74
5.333	astrocyte3	51	54	1.059	1.307	0.216	control3	67
23.915	astrocyte1	62	70	1.129	2.355	1.795	control1	19
20.043	astrocyte2	77	93	1.208	2.83	2.153	control2	26
18.16							control1	42
21.915							control2	51
14.274							control3	42
**9.831846**						**1.144264**		
**0.843535**						**0.13879**		
	astrocyte1	29	394	13.586	8.763	8.117	control1	20
	astrocyte2	34	732	21.529	13.377	12.272	control2	13
	astrocyte3	16	228	14.25	6.074	4.563	control3	21
	astrocyte4	28	389	13.893	8.228	7.16	control4	21
	astrocyte5	36	370	10.278	5.365	4.645	control5	23
	astrocyte6	27	484	17.926	13.246	12.154	control6	14
	astrocyte7	34	382	11.235	4.955	4.573	control7	16
	astrocyte8	21	425	20.238	7.358	6.83	control8	13
	astrocyte9	42	531	12.643	5.35	4.387	control9	28
	astrocyte10	26	410	15.769	8.613	8.329	control10	12
	astrocyte1	30	708	23.6	16.113	15.342	control1	9
	astrocyte2	35	794	22.686	18.013	17.381	control2	20
	astrocyte3	30	567	18.9	15.82	15.457	control3	15
	astrocyte4	28	1072	38.286	22.955	22.713	control4	6
	astrocyte5	39	564	14.462	15.153	14.632	control5	23
	astrocyte6	33	347	10.515	7.29	6.663	control6	18
	astrocyte7	33	781	23.667	15.484	14.655	control7	23
	astrocyte8	24	554	23.083	14.656	14.457	control8	10
	astrocyte9	33	668	20.242	10.467	10.264	control9	9
	astrocyte1	31	544	17.548	13.992	13.745	control1	12
	astrocyte2	21	396	18.857	9.602	9.377	control2	9
	astrocyte3	39	611	15.667	12.075	11.591	control3	20
	astrocyte4	25	668	26.72	12.384	11.903	control4	11
	astrocyte5	24	1103	45.958	26.187	25.773	control5	11

	astrocyte6	24	565	23.542	15.556	14.904	control6	11
	astrocyte7	23	545	23.696	17.085	16.595	control7	17
	astrocyte8	33	569	17.242	13.58	13.245	control8	11
	astrocyte1	33	711	21.545	22.514	22.042	control1	9
	astrocyte2	23	568	24.696	18.514	18.225	control2	13
	astrocyte3	29	900	31.034	20.134	19.487	control3	20
	astrocyte4	27	816	30.222	22.442	21.773	control4	22
	astrocyte5	33	877	26.576	24.402	23.475	control5	18
	astrocyte6	30	700	23.333	16.151	15.626	control6	12
	astrocyte7	21	767	36.524	19.31	18.482	control7	25
	astrocyte8	21	569	27.095	13.632	13.151	control8	13
	astrocyte9	33	513	15.545	9.646	9.257	control9	17
					**mean**	**13.42347**		
					**SEM**	**0.961348**		
15.042							control1	7
24.565							control2	19
17.223							control3	19
12.331							control4	11
9.734							control5	44
10.658							control6	32
21.354							control7	36
9.927							control1	44
9.564							control2	81
9.189							control3	68
19.214							control4	34
22.053							control5	67
10.469							control6	39
16.482							control7	34
10.641							control1	26
13.019							control2	41
7.916							control3	31
12.362							control4	42
7.594							control1	24
9.989							control2	14
17.877							control1	7
12.778							control1	84
13.205							control1	317
22.125							control2	212

9.864							control1	447
12.42							control2	366
10.586							control1	193
9.392							control2	325
15.029							control3	188
8.179							control1	366
15.827							control2	410
19.473							control3	276
17.908							control1	398
22.374	astrocyte1	36	59	1.639	1.489	0.089	control1	126
19.702	astrocyte2	29	58	2	1.452	0.315	control2	68
	astrocyte3	33	107	3.242	2.73	2.559	control3	23
	astrocyte4	16	22	1.375	0.772	0.753	control4	1
	astrocyte5	32	67	2.094	2.381	1.947	control5	26
23.092	astrocyte1	42	72	1.714	2.228	1.668	control1	48
19.622	astrocyte2	45	66	1.467	2.825	1.96	control2	127
17.281	astrocyte3	36	84	2.333	2.182	0.397	control3	87
	astrocyte4	34	62	1.824	1.31	0.809	control4	20
18.221	astrocyte1	4	7	1.75	0.11	-0.31	control1	26
23.928	astrocyte2	20	119	5.95	2.14	1.611	control2	66
	astrocyte3	18	96	5.333	2.133	2.055	control3	4
	astrocyte4	21	53	2.524	1.186	1.01	control4	6
	astrocyte5	11	15	1.364	0.328	0.224	control5	10
	astrocyte6	24	48	2	0.813	0.634	control6	4
	astrocyte7	20	64	3.2	1.137	0.713	control7	7
	astrocyte8	30	99	3.3	1.632	1.387	control8	7
	astrocyte9	23	47	2.043	0.846	0.095	control9	18
	astrocyte10	17	24	1.412	0.811	0.377	control10	20
	astrocyte11	38	69	1.816	1.388	1.208	control11	8
	astrocyte12	8	55	6.875	1.341	0.62	control12	19
	astrocyte13	4	5	1.25	0.131	-0.283	control13	17
22.318	astrocyte1	27	83	3.074	1.851	1.182	control1	105
15.282	astrocyte2	16	55	3.438	2.093	1.689	control2	49
	astrocyte3	33	102	3.091	2.003	1.814	control3	13
	astrocyte4	26	54	2.077	1.574	1.064	control4	23
8.276	astrocyte1	11	17	1.545	0.433	0.305	control1	19
12.217	astrocyte2	20	50	2.5	1.317	1.085	control2	11
	astrocyte3	12	31	2.583	0.967	0.846	control3	3

	astrocyte4	19	33	1.737	0.681	0.576	control4	7
	astrocyte5	40	161	4.025	3.933	3.918	control5	2
	astrocyte6	36	116	3.222	2.632	2.358	control6	15
	astrocyte7	10	22	2.2	0.477	0.315	control7	10
	astrocyte8	28	78	2.786	1.593	1.483	control8	5
	astrocyte9	10	15	1.5	0.42	0.375	control9	3
	astrocyte10	13	36	2.769	0.672	0.172	control10	18
13.358	astrocyte1	9	27	3	0.219	0.077	control1	13
21.058	astrocyte2	1	1	1	0.019	-0.298	control2	22
10.192	astrocyte3	2	2	1	0.028	-0.266	control3	24
18.974	astrocyte4	53	273	5.151	3.625	3.169	control4	32
12.557	astrocyte1	43	342	7.953	1.444	1.056	control1	170
24.362	astrocyte2	175	692	3.954	1.44	1.155	control2	108
22.845	astrocyte3	85	502	5.906	1.462	1.286	control3	83
	astrocyte4	222	649	2.923	1.71	1.499	control4	56
9.163	astrocyte1	94	163	1.734	0.433	-0.255	control1	384
8.284	astrocyte2	109	389	3.569	1.823	1.294	control2	280
7.407	astrocyte3	138	227	1.645	0.668	-0.225	control3	555
	astrocyte4	220	558	2.536	1.221	0.912	control4	94
8.721	astrocyte1	266	797	2.996	1.013	0.987	control1	41
14.642	astrocyte1	666	2205	3.311	2.121	1.355	control1	1079
**14.7833**					**mean**	**0.97532**		
**0.704597**					**SEM**	**0.128**		
	**Astrocytes**							
		**Count**	**Total area (pixel^2)**	**Average size (pixel^2)**	**%Size**			**Count**
34.845	astrocyte1	86	249	2.895	11.701	10.714	control1	49
35.716	astrocyte2	64	451	7.047	24.195	23.869	control2	18
35.295	astrocyte3	67	208	3.104	17.657	16.083	control3	59
48.46	astrocyte4	62	229	3.694	15.969	15.635	control4	20
56.355	astrocyte1	83	373	4.494	19.109	13.991	control1	132
50.98	astrocyte2	88	640	7.273	29.466	23.968	control2	44
36.758	astrocyte3	108	448	4.148	19.893	17.835	control3	65
40.176	astrocyte4	106	342	3.226	15.475	7.787	control4	140
41.166							control5	56
48.693	astrocyte1	85	316	3.718	14.535	9.644	control1	108
47.713	astrocyte2	63	150	2.381	9.434	4.324	control2	119
47.86	astrocyte3	32	96	3	6.818	2.076	control3	138
44.331	astrocyte4	99	328	3.313	18.283	14.307	control4	107

58.032								
35.096								
52.929	astrocyte1	79	244	3.089	16.053	14.642	control1	56
28.003	astrocyte2	117	240	2.051	7.762	5.994	control2	81
60.935	astrocyte3	139	386	2.777	14.71	12.713	control3	101
44.571	astrocyte1	84	161	1.917	8.905	6.558	control1	149
48.186	astrocyte2	37	44	1.189	2.193	0.591	control2	83
24.396	astrocyte3	82	127	1.549	6.615	4.914	control3	110
36.936	astrocyte1	77	262	3.403	7.009	6.331	control1	13
37.516	astrocyte2	103	433	4.204	13.161	13.113	control2	4
49.019	astrocyte3	48	82	1.708	3.814	3.549	control3	18
35.442							control4	6
36.856							control5	59
45.237	astrocyte1	46	110	2.391	4.729	3.854	control1	49
47.53	astrocyte2	77	116	1.506	5.55	4.618	control2	52
42.313	astrocyte3	71	141	1.986	6.932	6.175	control3	50
52.984	astrocyte4	68	369	5.426	14.784	12.427	control4	110
37.361	astrocyte5	71	239	3.366	10.025	9.268	control5	14
33.15	astrocyte1	97	214	2.206	7.771	5.159	control1	97
34.822	astrocyte2	89	229	2.573	7.628	6.861	control2	42
30.647	astrocyte3	67	172	2.567	6.178	2.556	control3	274
28.905	astrocyte4	53	87	1.642	3.708	2.102	control4	97
37.48	astrocyte1	64	105	1.641	6.191	3.443	control1	105
25.602	astrocyte2	41	57	1.39	2.681	0.938	control2	125
27.913	astrocyte3	92	213	2.315	8.237	7.498	control3	46
27.731	astrocyte4	65	133	2.046	5.542	3.726	control4	140
26.297	astrocyte5	92	136	1.478	5.743	4.604	control5	76
28.755	astrocyte1	44	59	1.341	2.679	0.525	control1	93
30.33	astrocyte2	25	35	1.4	2.103	0.726	control2	81
23.506	astrocyte3	17	18	1.059	1.042	-0.162	control3	105
22.734	astrocyte1	22	23	1.045	0.991	-0.547	control1	100
21.678	astrocyte2	7	7	1	0.395	-1.394	control2	121
53.803	astrocyte1	125	459	3.672	16.95	15.687	control1	60
46.052	astrocyte1	77	135	1.753	5.829	4.472	control1	59
29.609	astrocyte1	100	270	2.7	11.48	5.738	control1	160
23.779	astrocyte2	81	167	2.062	7.389	4.214	control2	241

23.406	astrocyte3	49	80	1.633	4.162	1.092	control3	126
31.443	astrocyte1	72	104	1.444	4.986	2.86	control1	116
30.227	astrocyte1	67	167	2.493	7.402	5.371	control1	101
37.979	astrocyte1	50	124	2.48	5.396	4.027	control1	58
25.805	astrocyte2	27	47	1.741	2.629	0.201	control2	61
32.561	astrocyte3	27	31	1.148	1.709	1.627	control3	6
40.547	astrocyte1	52	109	2.096	5.142	2.742	control1	95
22.877	astrocyte2	34	70	2.059	5.087	3.368	control2	131
28.728	astrocyte3	39	81	2.077	8.299	6.682	control3	81
32.886	astrocyte4	36	61	1.694	4.236	3.811	control4	43
43.829	astrocyte1	36	64	1.778	3.774	2.171	control1	118
33.508	astrocyte2	41	61	1.488	2.502	1.468	control2	62
24.893	astrocyte1	31	46	1.484	2.662	1.088	control1	121
37.871	astrocyte2	56	79	1.411	4.647	3.011	control2	93
38.683	astrocyte1	35	57	1.629	4.589	3.525	control1	71
**37.18322**					**mean**	**6.341864**		
**1.244315**					**SEM**	**0.759944**		
								**Count**
11.427							control1	23
27.93							control2	23
18.298							control3	23
15.434							control4	23
11.373							control5	23
8.175							control6	23
19.145							control1	31
3.281							control2	31
21.201							control3	31
8.641							control4	31
3.533							control5	31
14.242							control6	31
3.672							control1	42
7.126							control2	42
3.389							control3	42
2.368							control4	42

15.5	control5	42
18.809	control6	42
2.254	control7	42
8.113	control8	42
3.737	control1	33
4.324	control2	33
19.72	control3	33
18.583	control4	33
4.282	control1	24
4.383	control1	6
6.956	control2	6
14.21	control3	6
14.776	control1	27
8.933	control2	27
8.104	control3	27
11.733	control4	27
10.74	control5	27
11.021	control6	27
6.168	control7	27
8.602	control8	27
8.35	control1	35
15.955	control2	35
11.525	control3	35
10.522	control4	35
8.367	control5	35
8.068	control6	35
8.853	control7	35
4.617	control8	35
17.68	control9	35
9.625	control1	30
10.63	control2	30
6.86	control3	30
5.795	control4	30
8.031	control5	30
4.242	control6	30
14.942	control7	30
3.589	control1	18
6.126	control2	18
11.252	control3	18
3.528	control4	18

5.708	control1	12
3.374	control2	12
12.197	control3	12
16.964	control1	9
8.483	control2	9
10.568	control3	9
7.387	control4	9
9.853	control5	9
5.747	control6	9
12.478	control7	9
5.553	control8	9
9.679	control1	14
12.27	control2	14
17.963	control3	14
10.688	control4	14
6.9	control5	14
3.511	control6	14
6.545	control1	45
5.78	control2	45
4.813	control3	45
15.325	control4	45
8.934	control5	45
7.306	control6	45
3.533	control7	45
4.734	control1	32
10.99	control2	32
4.547	control3	32
3.475	control4	32
7.381	control5	32
4.356	control6	32
4.157	control7	32
3.295	control1	16
7.719	control2	16
3.394	control3	16
4.516	control4	16
4.564	control1	12
**9.058989**		
**0.539957**		

Astrocytes

	Count	Total area (pixel^2)	Average size (pixel^2)	%Size		Count
astrocyte1	4	5	1.25	1.742	control1	2
astrocyte2	1	5	5	1.592	control2	8
astrocyte3	3	6	2	2.532	control3	1
astrocyte4	5	7	1.4	2.8	control4	12
astrocyte5	1	1	1	0.355	control5	6
astrocyte1	6	9	1.5	2.528	control1	2
astrocyte2	7	13	1.857	2.68	control2	3
astrocyte3	7	16	2.286	4.02	control3	2
astrocyte4	6	9	1.5	4.265	control4	3
astrocyte5	11	16	1.455	3.071	control5	5
astrocyte1	12	19	1.583	3.711	control1	1
astrocyte2	8	14	1.75	3.182	control2	1
astrocyte3	6	16	2.667	3.383	control3	12
astrocyte4	3	5	1.667	1.558	control4	1
astrocyte5	4	7	1.75	1.961	control5	3
astrocyte1	18	31	1.722	4.429	control1	10
astrocyte2	8	8	1	1.097	control2	8
astrocyte3	12	14	1.167	1.947	control3	11
astrocyte4	8	14	1.75	2.205	control4	4
astrocyte5	9	9	1	1.362	control5	6
astrocyte1	7	13	1.857	3.234	control1	2
astrocyte2	11	19	1.727	3.792	control2	4
astrocyte3	4	8	2	2.122	control3	7
astrocyte4	7	10	1.429	2.703	control4	6
astrocyte5	6	12	2	3.061	control5	5
astrocyte1	12	27	2.25	5.444	control1	10
astrocyte2	8	13	1.625	2.857	control2	25
astrocyte3	8	21	2.625	3.933	control3	11
astrocyte4	13	27	2.077	4.072	control4	19
astrocyte5	28	49	1.75	7.778	control5	16
astrocyte1	4	6	1.5	1.504	control1	6
astrocyte2	8	9	1.125	1.903	control2	8
astrocyte3	15	31	2.067	4.913	control3	11
astrocyte4	19	22	1.158	2.781	control4	12
astrocyte5	10	17	1.7	3.257	control5	14
astrocyte1	12	17	1.417	2.515	control1	1
astrocyte2	12	25	2.083	3.852	control2	8

astrocyte3	15	27	1.8	4.592	control3	10
astrocyte4	13	16	1.231	2.516	control4	7
astrocyte5	8	11	1.375	2.345	control5	7
astrocyte1	5	5	1	1.202	control1	1
astrocyte2	5	5	1	1.65	control2	1
astrocyte3	2	4	2	1.194	control3	3
astrocyte4	3	4	1.333	1.223	control4	3
astrocyte5	2	2	1	0.483	control5	1
astrocyte1	4	4	1	1	control1	5
astrocyte2	3	3	1	0.767	control2	5
astrocyte3	1	1	1	0.243	control3	1
astrocyte4	4	4	1	1.183	control4	2
astrocyte5	3	3	1	0.89	control5	3
astrocyte1	4	11	2.75	3.548	control1	2
astrocyte2	5	7	1.4	2.115	control2	2
astrocyte3	3	3	1	0.811	control3	2
astrocyte4	2	3	1.5	0.958	control4	1
astrocyte5	2	2	1	0.651	control5	2
astrocyte1	5	7	1.4	1.663	control1	6
astrocyte2	16	32	2	6.426	control2	13
astrocyte3	8	14	1.75	3.474	control3	7
astrocyte4	10	14	1.4	2.692	control4	10
astrocyte5	12	18	1.5	4.196	control5	13
astrocyte1	20	29	1.45	3.092	control1	3
astrocyte2	7	14	2	1.852	control2	22
astrocyte3	4	4	1	0.474	control3	16
astrocyte4	7	10	1.429	1.222	control4	6
astrocyte5	1	1	1	0.142	control5	15
astrocyte1	10	12	1.2	1.382	control1	12
astrocyte2	5	9	1.8	0.98	control2	13
astrocyte3	23	28	1.217	3.851	control3	11
astrocyte4	7	10	1.429	1.433	control4	21
astrocyte5	21	28	1.333	2.887	control5	7
astrocyte1	1	2	2	0.615	control1	5
astrocyte2	4	6	1.5	1.415	control2	3
astrocyte3	3	4	1.333	1.108	control3	6
astrocyte4	4	4	1	1.286	control4	3
astrocyte5	5	5	1	1.319	control5	7
astrocyte1	14	25	1.786	2.934	control1	12

astrocyte2	19	35	1.842	4.667	control2	10
astrocyte3	15	22	1.467	2.781	control3	20
astrocyte4	30	50	1.667	6.676	control4	16
astrocyte5	5	13	2.6	2.808	control5	11
astrocyte1	6	9	1.5	1.09	control1	1
astrocyte2	11	12	1.091	1.818	control2	6
astrocyte3	4	6	1.5	0.817	control3	2
astrocyte4	9	10	1.111	1.445	control4	6
astrocyte5	6	7	1.167	1.014	control5	2
astrocyte1	6	8	1.333	1.141	control1	2
astrocyte2	6	8	1.333	1.233	control2	3
astrocyte3	9	11	1.222	1.91	control3	2
astrocyte4	11	25	2.273	4.521	control4	5
astrocyte5	4	5	1.25	0.711	control5	1
astrocyte1	4	10	2.5	1.058	control1	2
astrocyte2	8	10	1.25	1.445	control2	1
astrocyte3	7	7	1	1.028	control3	6
astrocyte4	14	22	1.571	2.308	control4	3
astrocyte5	5	7	1.4	1.148	control5	2
astrocyte1	12	16	1.333	2.222	control1	16
astrocyte2	28	74	2.643	8.959	control2	13
astrocyte3	13	17	1.308	1.778	control3	15
astrocyte4	12	29	2.417	3.831	control4	10
astrocyte5	8	8	1	1.083	control5	15
astrocyte1	2	3	1.5	0.401	control1	1
astrocyte2	2	3	1.5	0.366	control2	5
astrocyte3	1	1	1	0.225	control3	1
astrocyte4	1	1	1	0.149	control4	1
astrocyte5	1	1	1	0.177	control5	3
astrocyte1	2	2	1	0.236	control1	5
astrocyte2	1	1	1	0.097	control2	2
astrocyte3	3	3	1	0.285	control3	4
astrocyte4	6	8	1.333	1.153	control4	2
astrocyte5	4	4	1	0.496	control5	1
astrocyte1	5	7	1.4	1.01	control1	3
astrocyte2	9	12	1.333	1.805	control2	8
astrocyte3	3	4	1.333	0.658	control3	11
astrocyte4	5	5	1	0.571	control4	3
astrocyte5	10	15	1.5	1.765	control5	7

astrocyte1	3	5	1.667	0.779	control1	6
astrocyte2	2	2	1	0.337	control2	1
astrocyte3	14	17	1.214	2.391	control3	5
astrocyte4	3	3	1	0.457	control4	4
astrocyte5	6	6	1	0.957	control5	4
astrocyte1	1	1	1	0.549	control1	2
astrocyte2	3	5	1.667	2.538	control2	7
astrocyte3	3	5	1.667	2.577	control3	4
astrocyte4	3	7	2.333	4.142	control4	2
astrocyte5	1	1	1	0.671	control5	7
astrocyte1	9	11	1.222	1.94	control1	8
astrocyte2	8	9	1.125	1.278	control2	18
astrocyte3	6	7	1.167	1.082	control3	19
astrocyte4	20	32	1.6	3.292	control4	18
astrocyte5	10	10	1	1.645	control5	22
astrocyte1	4	7	1.75	1.301	control1	7
astrocyte2	9	9	1	1.957	control2	6
astrocyte3	10	15	1.5	2.4	control3	5
astrocyte4	4	5	1.25	1.16	control4	3
astrocyte5	12	19	1.583	1.87	control5	2
astrocyte1	10	20	2	3.683	control1	11
astrocyte2	26	66	2.538	9.231	control2	15
astrocyte3	4	5	1.25	1.211	control3	29
astrocyte4	11	36	3.273	6.844	control4	21
astrocyte5	9	11	1.222	2.007	control5	18
astrocyte1	24	61	2.542	8.402	control1	18
astrocyte2	15	33	2.2	6.145	control2	15
astrocyte3	11	19	1.727	3.14	control3	20
astrocyte4	25	36	1.44	5.31	control4	27
astrocyte5	14	20	1.429	2.886	control5	34
astrocyte1	8	16	2	5.387	control1	13
astrocyte2	9	20	2.222	6.645	control2	8
astrocyte3	5	6	1.2	1.749	control3	4
astrocyte4	6	7	1.167	2.28	control4	3
astrocyte5	8	8	1	2.36	control5	3
astrocyte1	5	8	1.6	2.111	control1	4
astrocyte2	3	4	1.333	0.971	control2	3
astrocyte3	2	3	1.5	0.732	control3	4
astrocyte4	8	8	1	2.116	control4	5
astrocyte5	1	1	1	0.297	control5	2

astrocyte1	4	5	1.25	1.37	control1	5
astrocyte2	4	4	1	1.227	control2	3
astrocyte3	8	11	1.375	2.709	control3	7
astrocyte4	10	22	2.2	5.641	control4	6
astrocyte5	7	15	2.143	3.659	control5	5
astrocyte1	9	15	1.667	3.713	control1	4
astrocyte2	13	28	2.154	5.773	control2	17
astrocyte3	7	25	3.571	5.952	control3	8
astrocyte4	10	16	1.6	4.598	control4	6
astrocyte5	6	15	2.5	4.286	control5	9
astrocyte1	23	40	1.739	5.202	control1	22
astrocyte2	10	15	1.5	2.03	control2	27
astrocyte3	9	12	1.333	1.376	control3	29
astrocyte4	8	15	1.875	1.469	control4	6
astrocyte5	12	14	1.167	1.41	control5	23
astrocyte1	5	7	1.4	2.602	control1	6
astrocyte2	7	19	2.714	6.441	control2	4
astrocyte3	2	2	1	0.948	control3	4
astrocyte4	7	14	2	5.091	control4	3
astrocyte5	9	15	1.667	6.944	control5	7
astrocyte1	6	6	1	1.23	control1	32
astrocyte2	16	21	1.312	4.046	control2	29
astrocyte3	13	22	1.692	3.612	control3	11
astrocyte4	10	17	1.7	3.346	control4	30
astrocyte5	14	32	2.286	5.031	control5	9
astrocyte1	12	16	1.333	2.207	control1	21
astrocyte2	9	14	1.556	2.009	control2	11
astrocyte3	7	10	1.429	2	control3	17
astrocyte4	9	10	1.111	1.541	control4	13
astrocyte5	7	10	1.429	1.789	control5	19
astrocyte1	15	30	2	5.747	control1	12
astrocyte2	12	18	1.5	3.12	control2	25
astrocyte3	10	21	2.1	4.403	control3	9
astrocyte4	6	6	1	1.63	control4	2
astrocyte5	8	12	1.5	2.673	control5	1
astrocyte1	11	13	1.182	2.826	control1	14
astrocyte2	15	31	2.067	5.536	control2	12
astrocyte3	6	12	2	2.521	control3	11
astrocyte4	13	20	1.538	3.252	control4	12

astrocyte5	7	8	1.143	1.354	control5	17
astrocyte1	2	2	1	0.402	control1	11
astrocyte2	12	22	1.833	3.86	control2	6
astrocyte3	17	37	2.176	6.801	control3	3
astrocyte4	18	31	1.722	5.429	control4	5
astrocyte5	13	18	1.385	3.364	control5	4
astrocyte1	3	3	1	0.617	control1	7
astrocyte2	4	4	1	0.871	control2	8
astrocyte3	1	1	1	0.13	control3	3
astrocyte4	7	8	1.143	1.616	control4	2
astrocyte5	3	3	1	0.472	control5	10

Control		
Total area (pixel^2)	Average size (pixel^2)	%Size
3	1	0.026
6	2	0.042
4	2	0.039
5	1.25	0.051
10	2.5	0.119
17	1.545	0.341
3	1.5	0.06
36	3	0.653
13	1.444	0.261
32	2.133	0.57
47	3.133	0.652
2	1	0.046
28	3.111	0.38
6	2	0.1
30	3.333	0.504
62	2.385	0.728
27	5.4	0.351
235	6.528	0.48
94	3.615	0.317
322	2.205	0.503
318	4.297	0.803
262	3.316	0.712
196	5.158	0.416
62	1.033	2.188
97	1.406	3.647
54	1.174	1.723
35	1.094	1.059
55	1.1	1.645
81	1.125	1.325
46	1.045	0.948

73	1.106	1.579
88	1.158	1.562
65	1.204	1.739
14	1	0.768
25	1.042	0.872
36	1	0.693
11	1	0.253
19	1	0.595
7	1	0.29
32	1.185	0.894
46	1.095	0.516
86	1.323	0.956
76	1.056	0.926
187	1.087	1.783
156	1.156	2.746
137	1.114	2.507
125	1.168	1.7
19	1.462	0.636
12	1.091	0.226
16	1.231	0.266
44	1.222	0.692
15	1.154	0.317
3	1	0.059
10	1	0.166
9	1	0.241
3	1	0.052
2	1	0.034
8	1	0.123
5	1	0.083
18	1	0.636
42	1.2	1.302
54	1.102	1.538
51	1.062	1.48
30	1.034	0.83

122	1.151	2.128
82	1.038	1.173
66	1.048	1.02
49	1.043	1.235
97	1.228	1.561
63	1.033	0.931
68	1.079	1.373
53	1.06	0.748
90	1.216	1.293
73	1.09	1.091
19	1	0.56
26	1	0.677
48	1.143	0.582
57	1.118	0.646
43	1.024	0.629
45	2.25	0.646
71	5.462	1.105
117	5.571	1.511
118	5.619	1.068
71	3.087	0.72
96	6.857	1.092
40	2.5	0.382
51	3.923	0.528
93	3.321	0.963
26	2.167	0.284
50	5.556	0.771
70	3.5	0.632
33	2.2	0.363
17	2.833	0.242
52	2.261	0.521
55	3.056	0.627
103	4.478	0.829
18	1.8	0.199
23	2.556	0.203
22	1.833	0.247
16	1.778	0.225
52	2.6	0.484
41	3.727	0.481
38	3.455	0.414

62	5.636	0.652
56	3.294	0.49
36	3.273	0.335
37	4.111	0.472
30	2.308	0.289
82	4.1	0.647
96	4.364	0.669
91	5.056	0.927
53	4.417	0.525
97	3.88	0.828
73	5.615	0.481
62	3.647	0.389
30	4.286	0.217
72	3.789	0.504
51	2.684	0.294
42	3.818	0.192
104	2.364	0.504
80	2.5	0.569
67	1.861	0.309
158	3.591	0.601
161	1.988	0.651
199	2.926	1.082
74	2.176	0.502
173	2.582	0.725
120	3.077	0.856
105	3.088	0.654
97	3.731	0.552
126	3.073	0.71
76	2.452	0.437
99	2.357	0.317
41	1.708	0.259
24	1.714	0.151
14	2	0.093
486	5.786	0.408
795	2.508	1.064
627	2.958	0.972

1986	4.443	2.218
1279	3.495	1.373
944	4.891	0.93
865	2.662	1.131
757	4.027	1.25
1297	3.544	0.949
968	2.361	0.702
858	3.109	0.536
937	2.354	0.558
252	2	1.4
124	1.824	1.137
32	1.391	0.171
1	1	0.019
44	1.692	0.434
67	1.396	0.56
178	1.402	0.865
175	2.011	1.785
28	1.4	0.501
76	2.923	0.42
130	1.97	0.529
7	1.75	0.078
13	2.167	0.176
16	1.6	0.104
19	4.75	0.179
22	3.143	0.424
12	1.714	0.245
53	2.944	0.751
38	1.9	0.434
16	2	0.18
66	3.474	0.721
34	2	0.414
225	2.143	0.669
95	1.939	0.404
16	1.231	0.189
46	2	0.51
21	1.105	0.128
41	3.727	0.232
9	3	0.121

7	1	0.105
2	1	0.015
23	1.533	0.274
11	1.1	0.162
6	1.2	0.11
3	1	0.045
34	1.889	0.5
25	1.923	0.142
58	2.636	0.317
61	2.542	0.294
86	2.688	0.456
391	2.3	0.388
250	2.315	0.285
119	1.434	0.176
88	1.571	0.211
743	1.935	0.688
545	1.946	0.529
1172	2.112	0.893
137	1.457	0.309
50	1.22	0.026
1728	1.601	0.766

Control

Total area (pixel^2)	Average size (pixel^2)	%Size
64	1.306	0.987
18	1	0.326
109	1.847	1.574
21	1.05	0.334
229	1.735	5.118
127	2.886	5.498
87	1.338	2.058
254	1.814	7.688
131	2.339	4.229
171	1.583	4.891
190	1.597	5.11
217	1.572	4.742
162	1.514	3.976

66	1.179	1.411
92	1.136	1.768
117	1.158	1.997
179	1.201	2.347
98	1.181	1.602
126	1.145	1.701
24	1.846	0.678
4	1	0.048
24	1.333	0.265
6	1	0.086
71	1.203	0.796
53	1.082	0.875
72	1.385	0.932
67	1.34	0.757
144	1.309	2.357
31	2.214	0.757
126	1.299	2.612
49	1.167	0.767
365	1.332	3.622
145	1.495	1.606
159	1.514	2.748
143	1.144	1.743
56	1.217	0.739
174	1.243	1.816
100	1.316	1.139
120	1.29	2.154
93	1.148	1.377
122	1.162	1.204
121	1.21	1.538
161	1.331	1.789
95	1.583	1.263
81	1.373	1.357
271	1.694	5.742
335	1.39	3.175

201	1.595	3.07
133	1.147	2.126
117	1.158	2.031
68	1.172	1.369
94	1.541	2.428
6	1	0.082
120	1.263	2.4
158	1.206	1.719
104	1.284	1.617
45	1.047	0.425
141	1.195	1.603
80	1.29	1.034
141	1.165	1.574
106	1.14	1.636
80	1.127	1.064

Control		
**Total area (pixel^2)**	**Average size (pixel^2)**	**%Size**
82	3.565	1.134
82	3.565	1.134
82	3.565	1.134
82	3.565	1.134
82	3.565	1.134
82	3.565	1.134
66	2.129	0.441
66	2.129	0.441
66	2.129	0.441
66	2.129	0.441
66	2.129	0.441
66	2.129	0.441
95	2.262	1.16
95	2.262	1.16
95	2.262	1.16
95	2.262	1.16

95	2.262	1.16
95	2.262	1.16
95	2.262	1.16
95	2.262	1.16
109	3.303	1.234
109	3.303	1.234
109	3.303	1.234
109	3.303	1.234
54	2.25	0.473
37	6.167	0.621
37	6.167	0.621
37	6.167	0.621
72	2.667	0.769
72	2.667	0.769
72	2.667	0.769
72	2.667	0.769
72	2.667	0.769
72	2.667	0.769
72	2.667	0.769
72	2.667	0.769
113	3.229	1.449
113	3.229	1.449
113	3.229	1.449
113	3.229	1.449
113	3.229	1.449
113	3.229	1.449
113	3.229	1.449
113	3.229	1.449
113	3.229	1.449
112	3.733	1.194
112	3.733	1.194
112	3.733	1.194
112	3.733	1.194
112	3.733	1.194
112	3.733	1.194
112	3.733	1.194
65	3.611	0.583
65	3.611	0.583
65	3.611	0.583
65	3.611	0.583

39	3.25	0.365
39	3.25	0.365
39	3.25	0.365
40	4.444	0.257
40	4.444	0.257
40	4.444	0.257
40	4.444	0.257
40	4.444	0.257
40	4.444	0.257
40	4.444	0.257
40	4.444	0.257
76	5.429	0.573
76	5.429	0.573
76	5.429	0.573
76	5.429	0.573
76	5.429	0.573
76	5.429	0.573
80	1.778	0.998
80	1.778	0.998
80	1.778	0.998
80	1.778	0.998
80	1.778	0.998
80	1.778	0.998
80	1.778	0.998
108	3.375	0.795
108	3.375	0.795
108	3.375	0.795
108	3.375	0.795
108	3.375	0.795
108	3.375	0.795
108	3.375	0.795
46	2.875	0.278
46	2.875	0.278
46	2.875	0.278
46	2.875	0.278
16	1.333	0.194

Control

Total area (pixel^2)	Average size (pixel^2)	%Size
2	1	0.515
12	1.5	2.516
1	1	0.187
14	1.167	2.258
8	1.333	1.363
2	1	0.366
8	2.667	1.124
2	1	0.269
4	1.333	0.622
6	1.2	0.509
1	1	0.088
1	1	0.101
13	1.083	1.448
1	1	0.164
5	1.667	0.653
13	1.3	1.344
20	2.5	1.73
15	1.364	0.642
4	1	0.312
11	1.833	0.549
2	1	0.376
6	1.5	0.87
10	1.429	1.13
7	1.167	0.84
9	1.8	1.21
11	1.1	1.042
41	1.64	3.388
19	1.727	1.309
25	1.316	2.354
22	1.375	1.921
8	1.333	1.027
8	1	0.79
14	1.273	0.954
12	1	1.228
15	1.071	1.752
3	3	0.472
8	1	0.986

12	1.2	1.283
8	1.143	0.631
8	1.143	0.824
1	1	0.213
1	1	0.328
4	1.333	1.075
3	1	0.577
1	1	0.177
5	1	0.608
7	1.4	0.708
1	1	0.105
4	2	0.503
3	1	0.234
3	1.5	0.645
2	1	0.531
2	1	0.462
1	1	0.113
2	1	0.299
7	1.167	1.116
19	1.462	2.997
8	1.143	0.936
11	1.1	1.583
14	1.077	1.707
3	1	0.221
24	1.091	1.257
19	1.188	1.064
8	1.333	0.428
16	1.067	0.828
14	1.167	1.335
22	1.692	1.385
12	1.091	1.022
24	1.143	1.321
8	1.143	0.469
5	1	0.47
4	1.333	0.398
6	1	0.591
4	1.333	0.357
8	1.143	0.834
20	1.667	1.42

11	1.1	0.762
26	1.3	1.799
19	1.188	0.855
19	1.727	0.833
1	1	0.071
7	1.167	0.787
2	1	0.175
7	1.167	0.539
3	1.5	0.201
3	1.5	0.423
4	1.333	0.78
2	1	0.422
6	1.2	0.661
1	1	0.211
3	1.5	0.25
1	1	0.092
6	1	0.649
5	1.667	0.532
2	1	0.208
22	1.375	3.014
21	1.615	2.567
18	1.2	1.406
12	1.2	1.266
18	1.2	1.889
2	2	0.219
5	1	0.519
1	1	0.114
1	1	0.118
3	1	0.37
5	1	0.399
2	1	0.134
5	1.25	0.34
2	1	0.165
2	2	0.221
5	1.667	0.239
12	1.5	0.602
13	1.182	0.602
3	1	0.156
9	1.286	0.477

7	1.167	0.837
1	1	0.116
7	1.4	0.738
4	1	0.436
4	1	0.522
3	1.5	0.376
7	1	0.908
4	1	0.563
6	3	1.188
10	1.429	1.14
10	1.25	0.663
22	1.222	1.863
28	1.474	1.984
19	1.056	0.64
23	1.045	1.271
9	1.286	0.938
7	1.167	0.695
5	1	0.604
3	1	0.247
2	1	0.184
15	1.364	0.872
20	1.333	1.053
47	1.621	2.119
28	1.333	1.422
29	1.611	1.326
20	1.111	0.914
17	1.133	1.053
31	1.55	1.526
42	1.556	1.702
46	1.353	1.669
14	1.077	1.483
12	1.5	1.267
5	1.25	0.46
3	1	0.202
3	1	0.244
5	1.25	0.366
3	1	0.259
5	1.25	0.573
7	1.4	0.596
2	1	0.146

6	1.2	0.938
5	1.667	0.652
9	1.286	0.986
9	1.5	0.865
8	1.6	1.028
4	1	0.473
20	1.176	1.377
11	1.375	1.213
9	1.5	0.74
13	1.444	1.379
25	1.136	2.122
34	1.259	3.099
35	1.207	3.159
9	1.5	0.843
31	1.348	2.359
9	1.5	1.264
4	1	0.582
5	1.25	0.832
3	1	0.725
10	1.429	0.903
48	1.5	1.787
40	1.379	1.991
13	1.182	0.632
43	1.433	2.178
11	1.222	0.461
24	1.143	1.364
11	1	0.772
19	1.118	0.922
18	1.385	1.422
22	1.158	1.885
19	1.583	3.055
39	1.56	3.951
11	1.222	0.927
2	1	0.105
1	1	0.081
15	1.071	0.567
15	1.25	0.782
12	1.091	0.536
14	1.167	0.553

21	1.235	0.838
13	1.182	1.435
8	1.333	1.133
5	1.667	0.542
5	1	0.661
4	1	0.671
10	1.429	0.78
8	1	0.647
3	1	0.386
3	1.5	0.27
14	1.4	1.037

Regarding figure panels 1A1, 1A2, 1A3, and 6E, data were fetched from the Allen Brain map, specifically this link: https://portal.brain-map.org/atlases-and-data/rnaseq/human-multiple-cortical-areas-smart-seq
