# Supplementary figures and images for: Cell-specific expression of key mitochondrial enzymes limits OXPHOS in astrocytes of the adult human neocortex and hippocampal formation
Source: Commun Biol. 2024 Aug 24;7:1045. doi: 10.1038/s42003-024-06751-z (PMC11344819; doi:10.1038/s42003-024-06751-z)

DATASET Homo

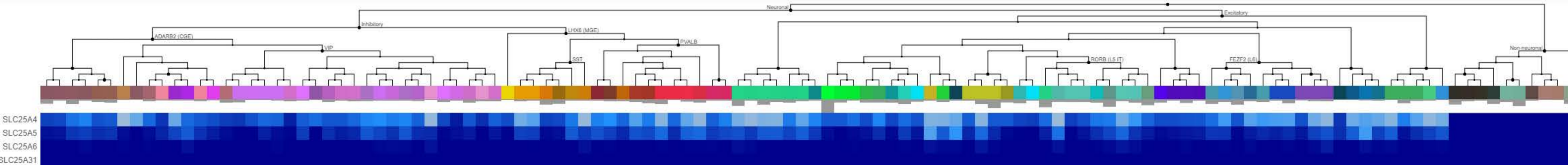

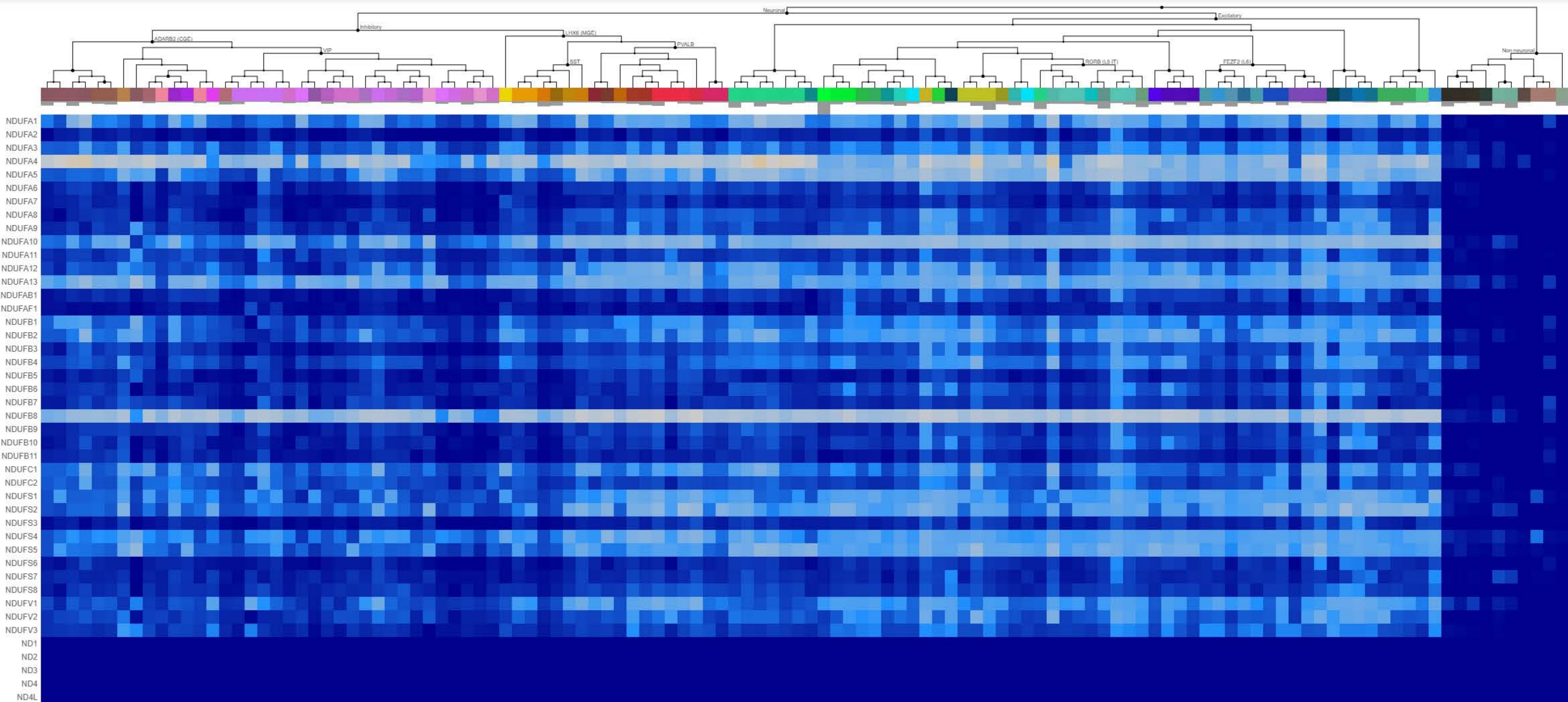

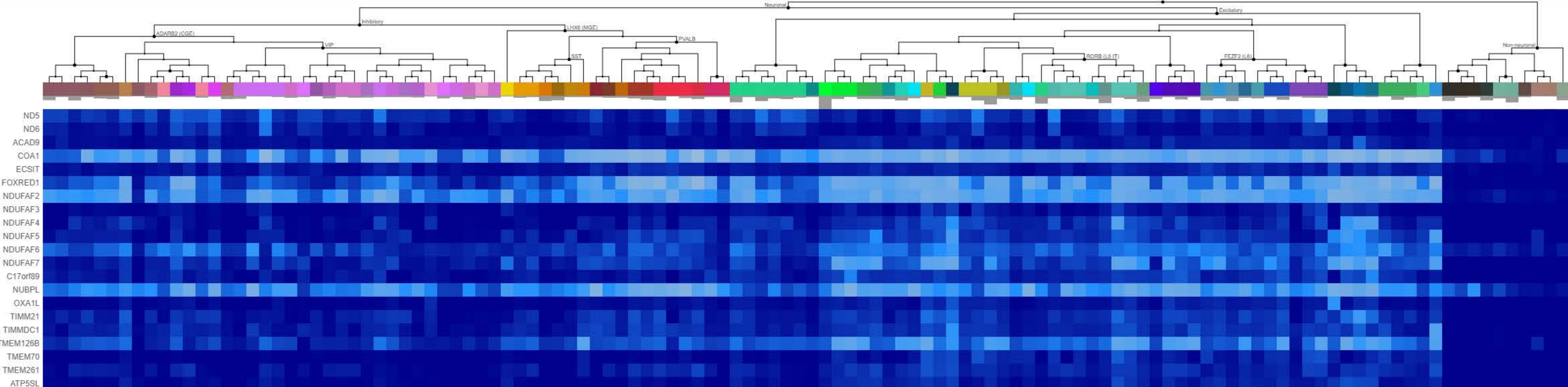

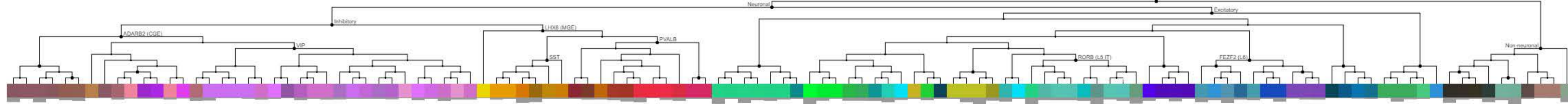

SDHA

SDHB

SDHC

SDHD

SDHAF1

SDHAF2

SDHAF3

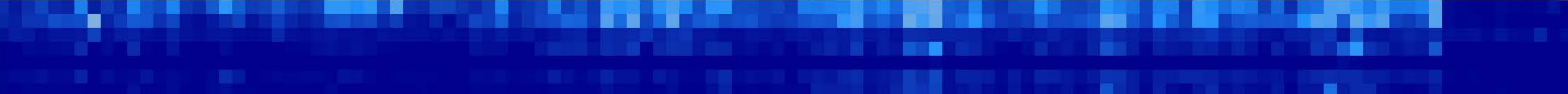

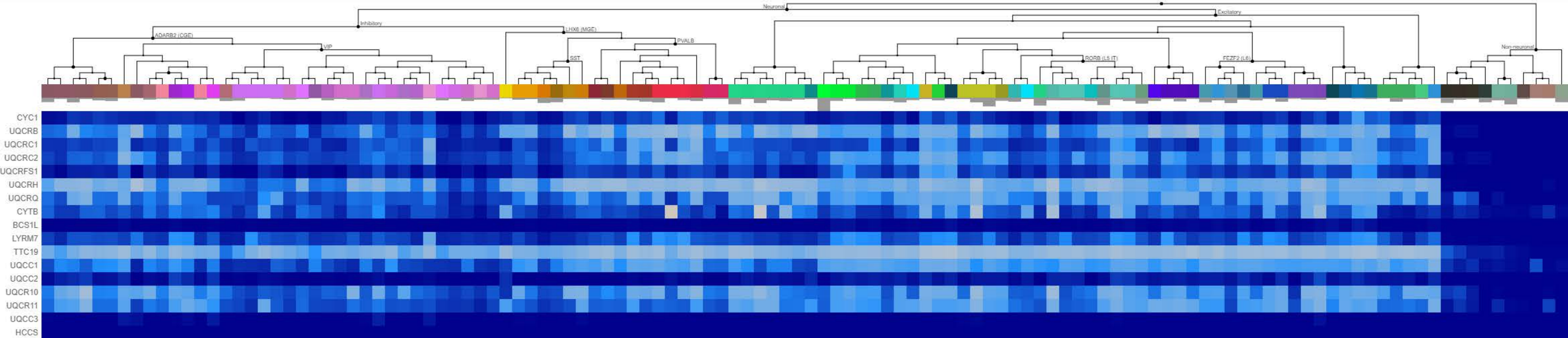

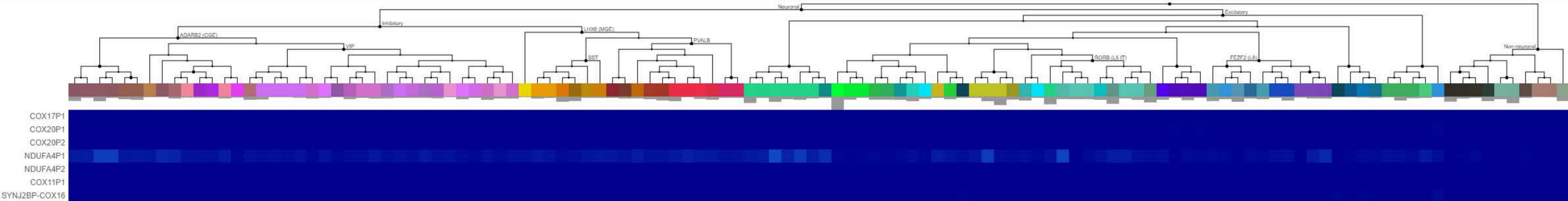

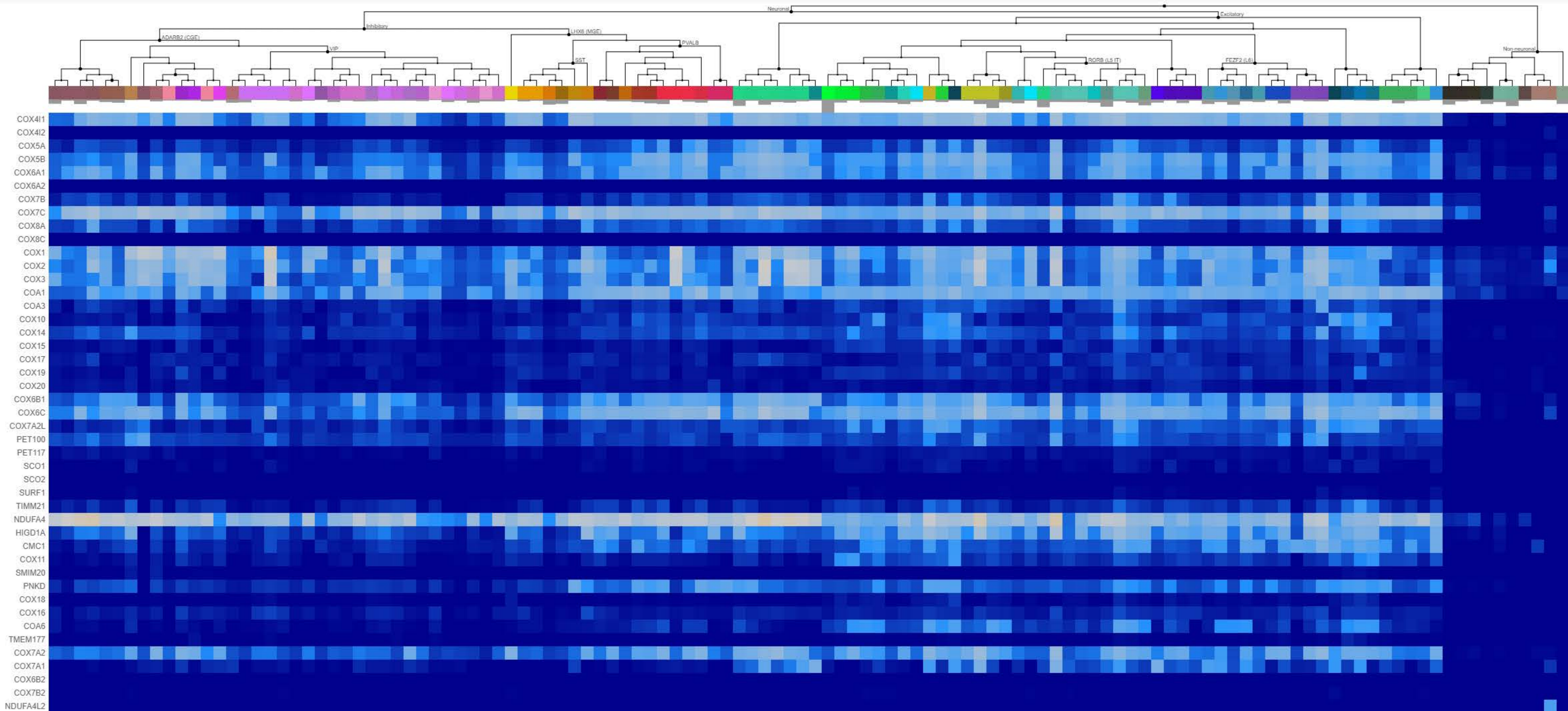

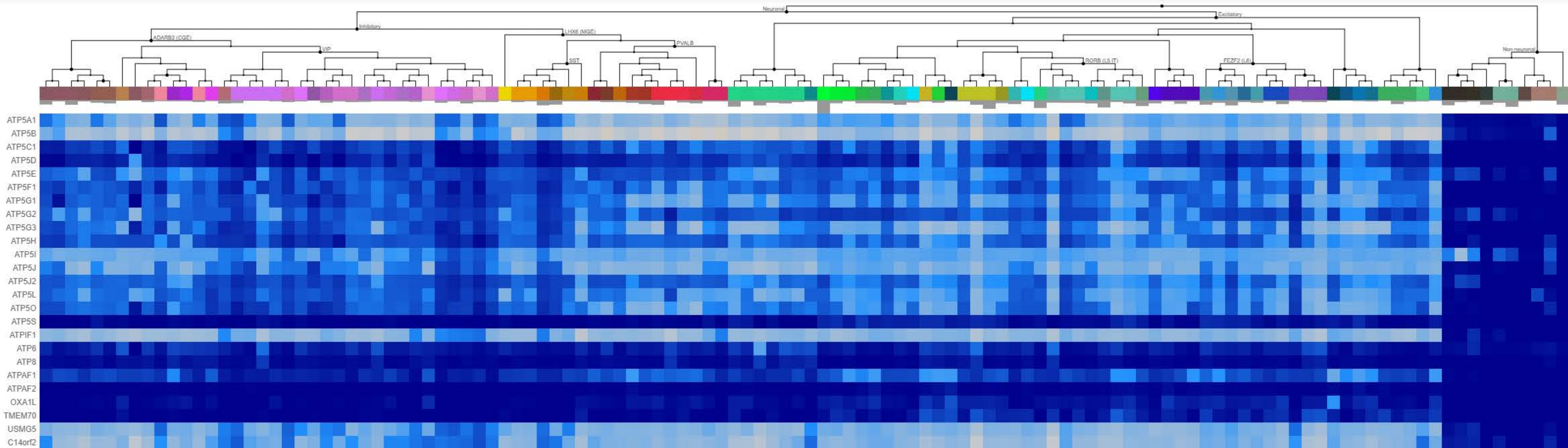

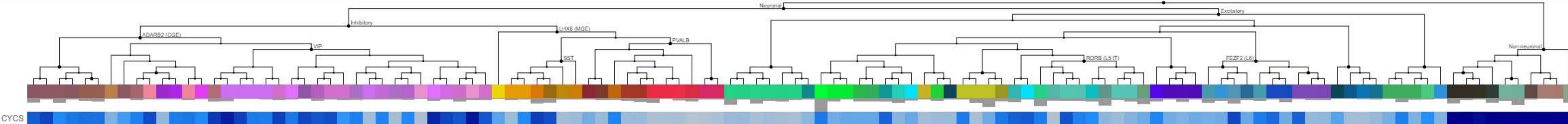

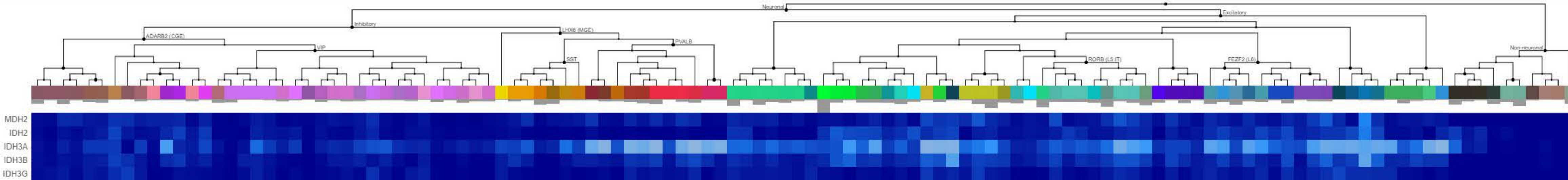

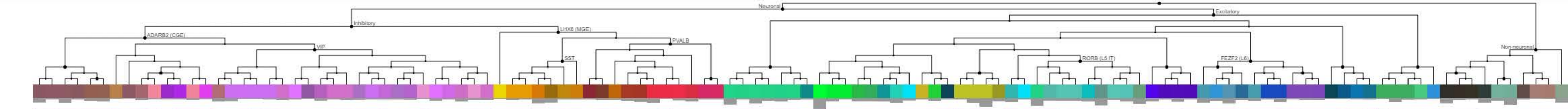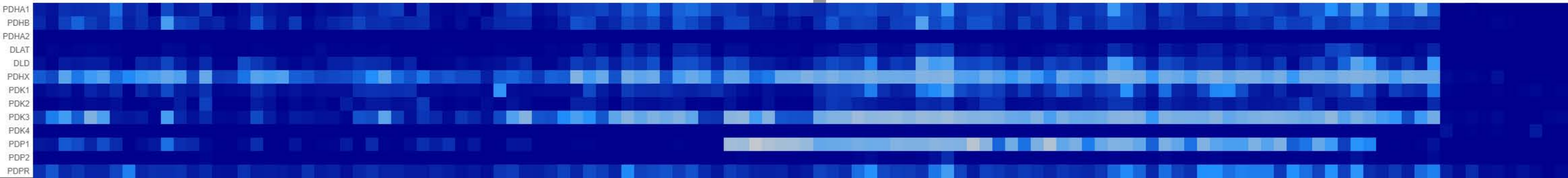

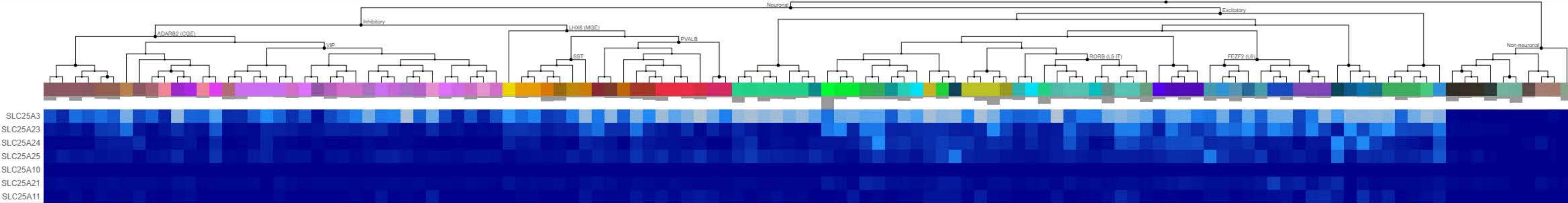

## DATASET Mus

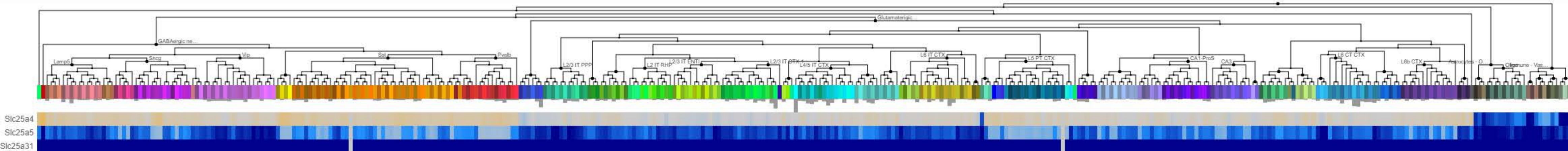

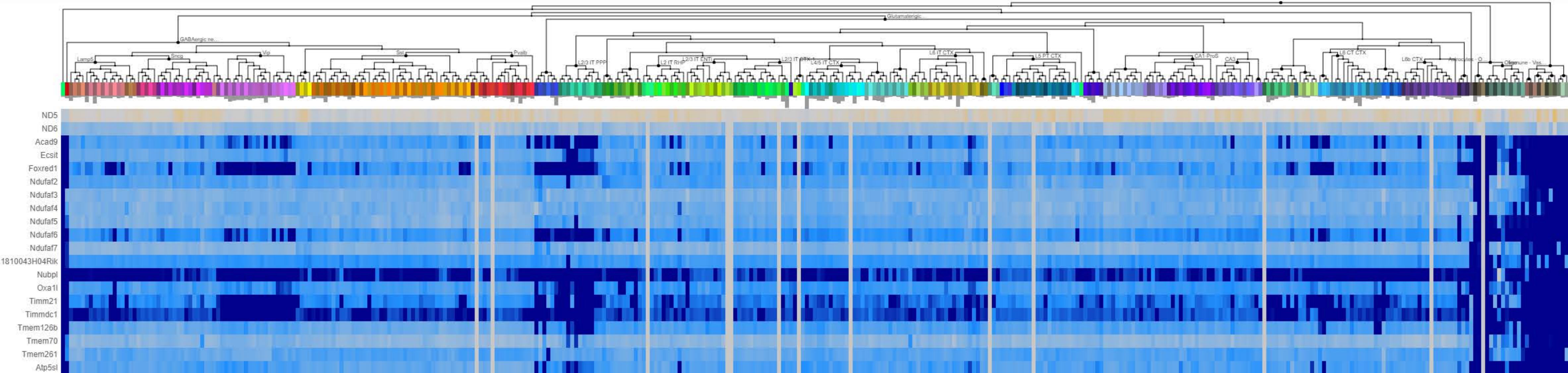

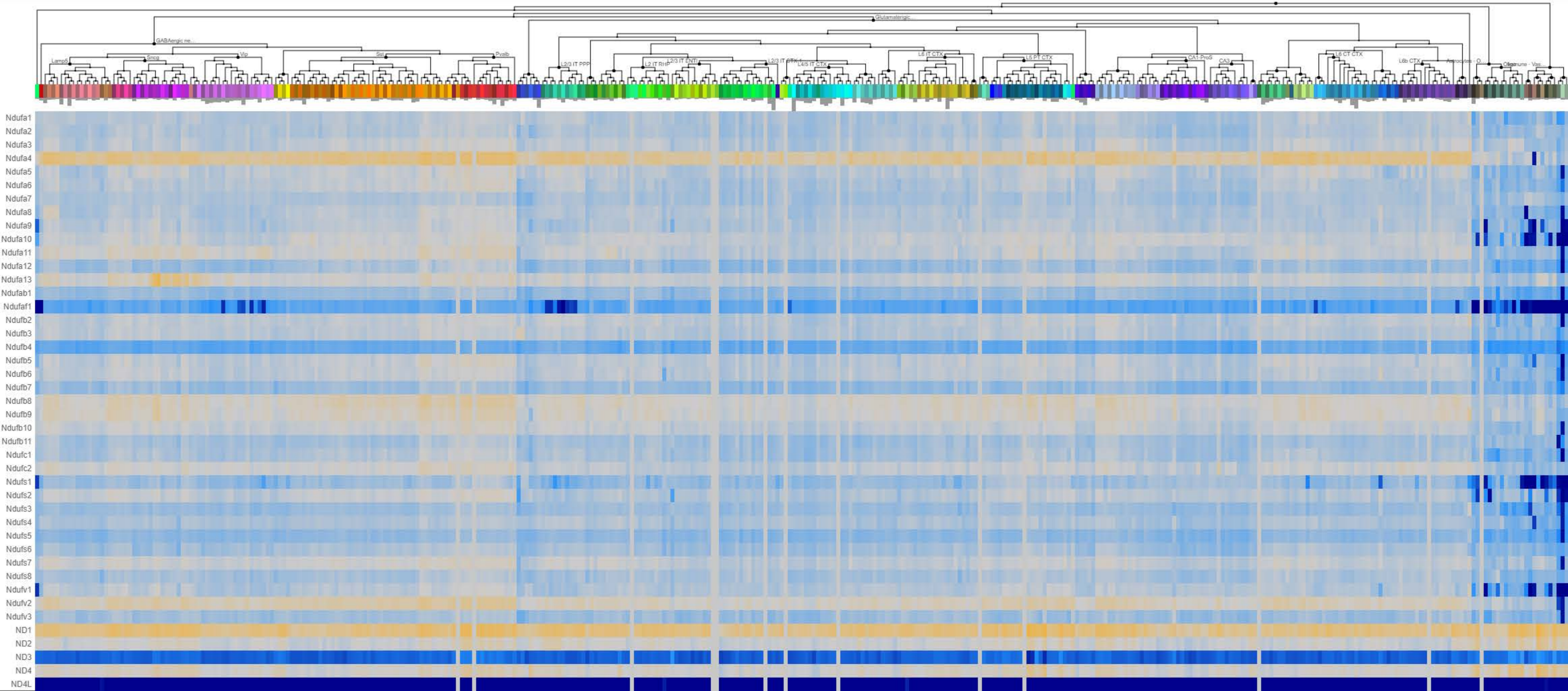

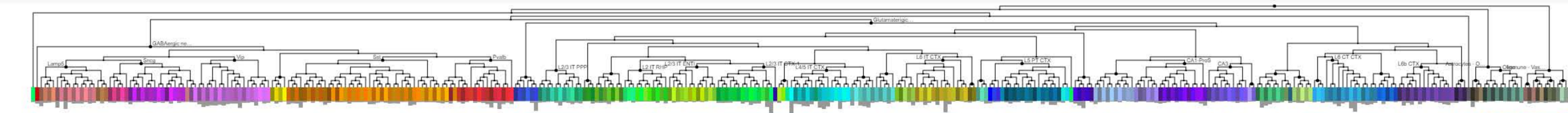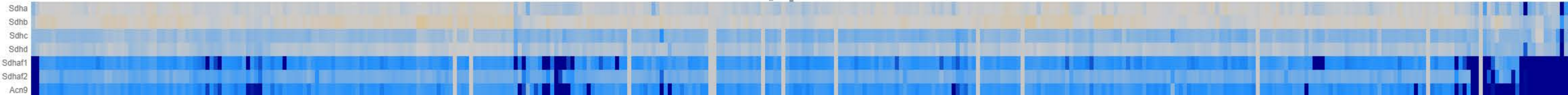

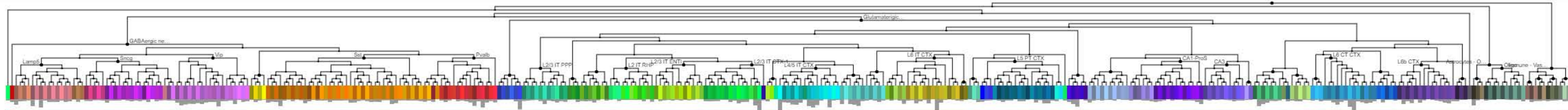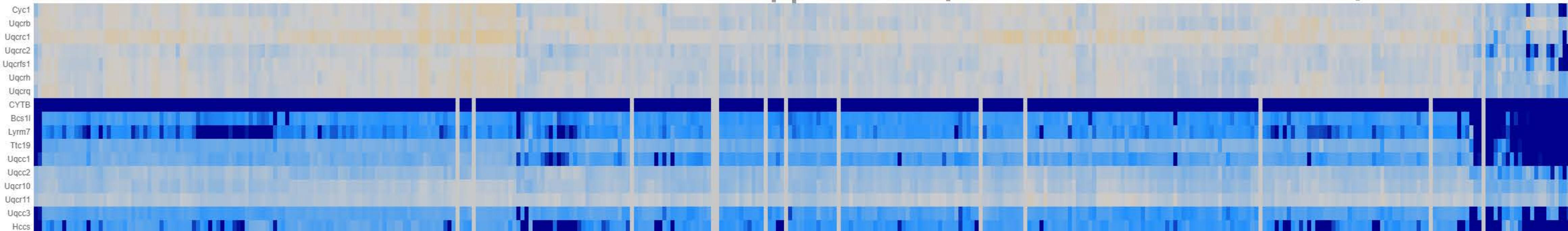

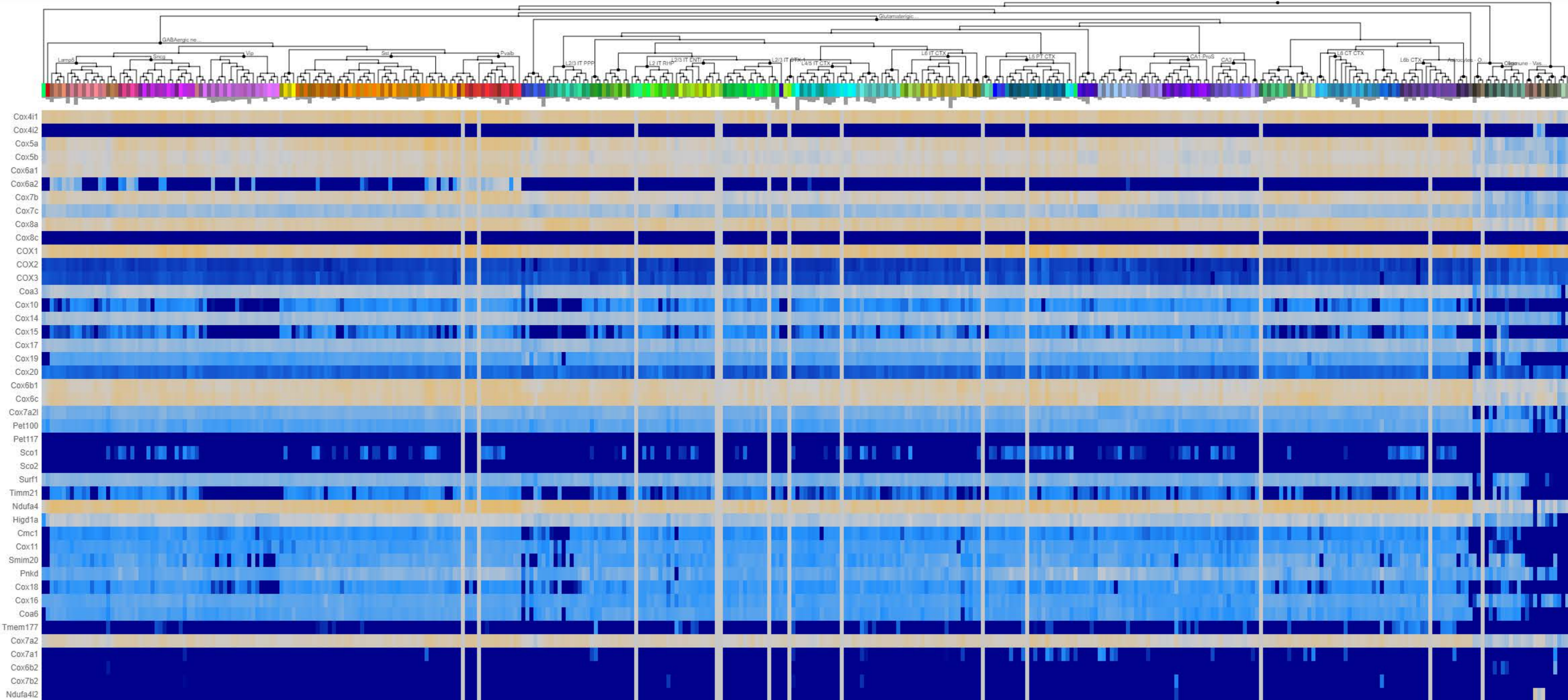

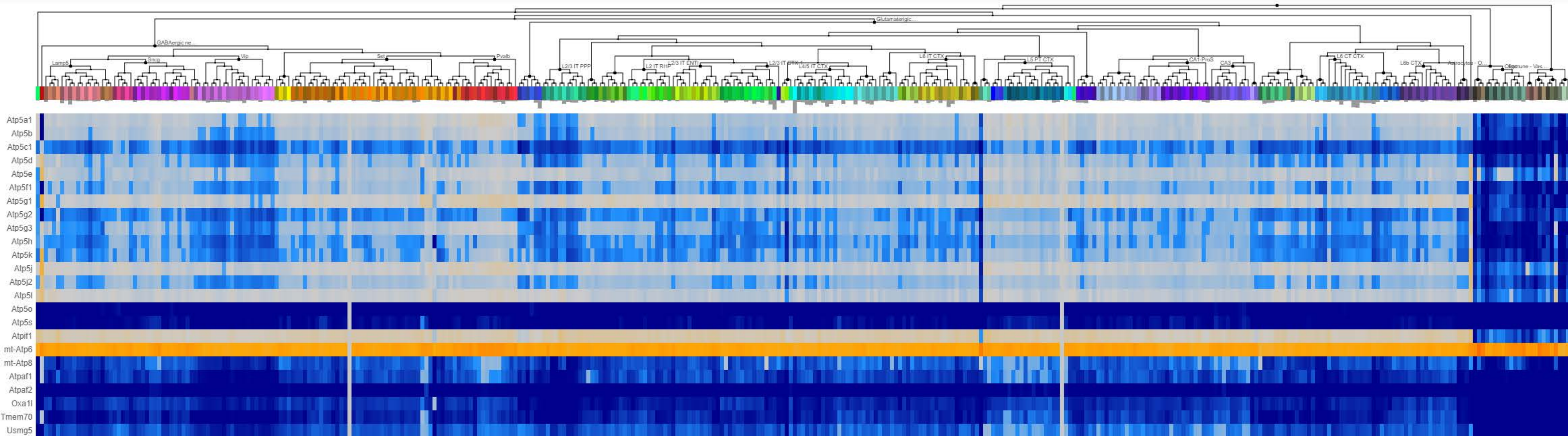

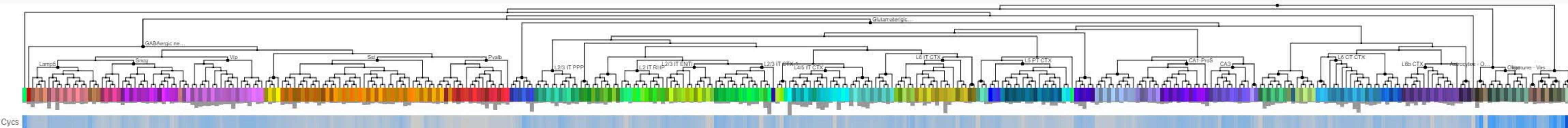

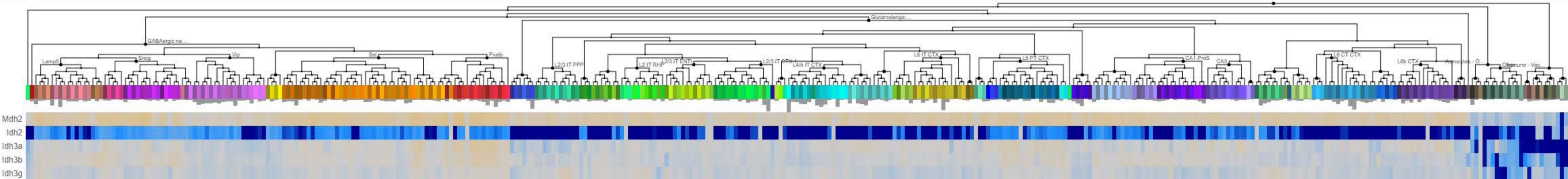

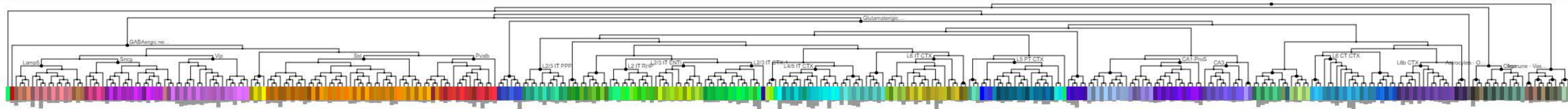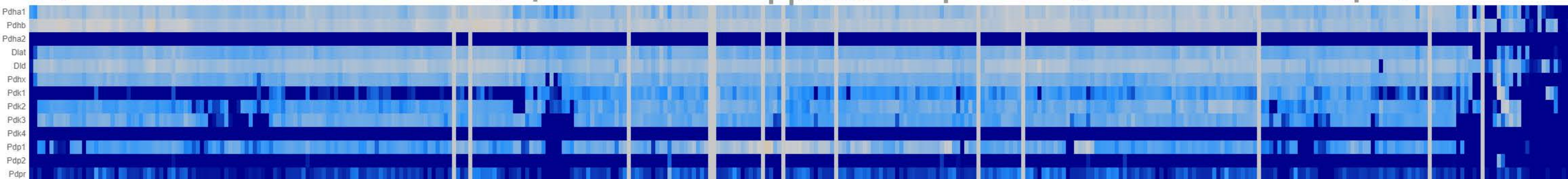

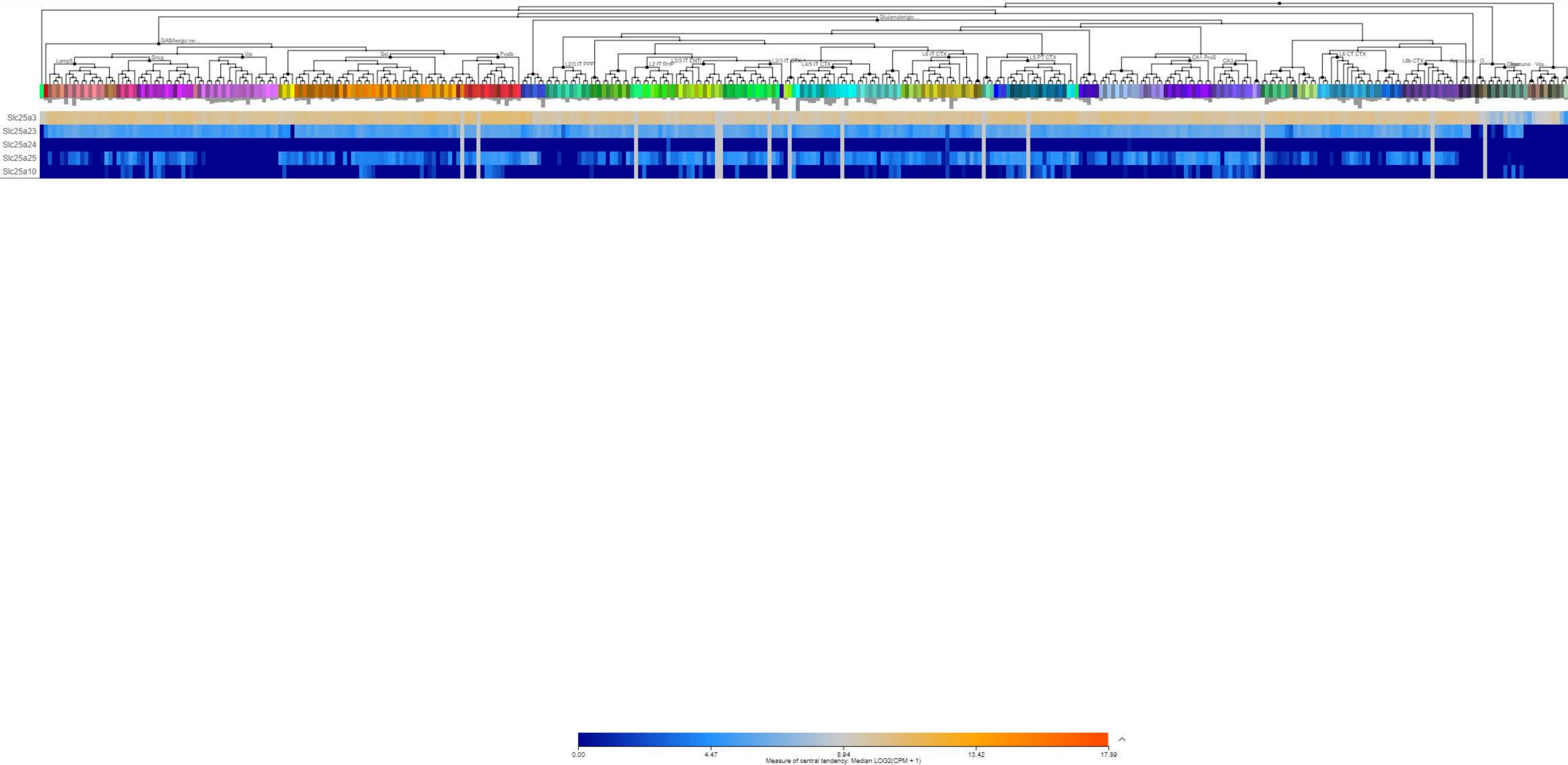

Supplement: Supplementary file 5 — Supplementary Data 2 [file 42003_2024_6751_MOESM5_ESM.pdf]
